# An automatic method for removing empty camera trap images using ensemble learning

**DOI:** 10.1002/ece3.7591

**Published:** 2021-05-02

**Authors:** Deng‐Qi Yang, Kun Tan, Zhi‐Pang Huang, Xiao‐Wei Li, Ben‐Hui Chen, Guo‐Peng Ren, Wen Xiao

**Affiliations:** ^1^ Department of Mathematics and Computer Science Dali University Dali China; ^2^ Institute of Eastern‐Himalaya Biodiversity Research Dali University Dali China; ^3^ Collaborative Innovation Center for the Biodiversity in the Three Parallel Rivers of China Dali China; ^4^ Data Security and Application Innovation Team Dali University Dali China

**Keywords:** artificial intelligence, camera trap images, convolutional neural networks, deep learning, ensemble learning

## Abstract

Camera traps often produce massive images, and empty images that do not contain animals are usually overwhelming. Deep learning is a machine‐learning algorithm and widely used to identify empty camera trap images automatically. Existing methods with high accuracy are based on millions of training samples (images) and require a lot of time and personnel costs to label the training samples manually. Reducing the number of training samples can save the cost of manually labeling images. However, the deep learning models based on a small dataset produce a large omission error of animal images that many animal images tend to be identified as empty images, which may lead to loss of the opportunities of discovering and observing species. Therefore, it is still a challenge to build the DCNN model with small errors on a small dataset. Using deep convolutional neural networks and a small‐size dataset, we proposed an ensemble learning approach based on conservative strategies to identify and remove empty images automatically. Furthermore, we proposed three automatic identifying schemes of empty images for users who accept different omission errors of animal images. Our experimental results showed that these three schemes automatically identified and removed 50.78%, 58.48%, and 77.51% of the empty images in the dataset when the omission errors were 0.70%, 1.13%, and 2.54%, respectively. The analysis showed that using our scheme to automatically identify empty images did not omit species information. It only slightly changed the frequency of species occurrence. When only a small dataset was available, our approach provided an alternative to users to automatically identify and remove empty images, which can significantly reduce the time and personnel costs required to manually remove empty images. The cost savings were comparable to the percentage of empty images removed by models.

## Graphical abstract

Based on deep convolutional neural networks and a smaller‐scalar dataset, we proposed an ensemble learning method using conservative strategies to automatically remove a part of empty images to save the personnel and time costs for manually removing empty images. Furthermore, we presented three schemes with different omission errors of animal images, which provided different alternatives for users with various tolerances for omission errors to remove different percentages of empty camera trap images automatically. Our experimental results showed that our schemes automatically removed 50.78%, 58.48%, and 77.51% of the empty images in the dataset when the omission errors were 0.70%, 1.13%, and 2.54%, respectively.

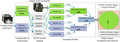

## 1 INTRODUCTION

Camera traps are widely used in ecological surveys due to their advantages of noninvasiveness, noninterference, and low cost. Camera traps deployed in the wild can help ecologists study species richness (Forsyth et al., 2019), population and distribution (Webb et al., 2016), habitat use (Dertien et al., 2017; Thorne et al., 2017), etc. It provides better decision support for ecological monitoring and protection. Camera traps usually produce a large number of images, and the proportion of empty images not containing animals can be overwhelming (Diaz‐Pulido & Payan, 2011; Swanson et al., 2015; Willi et al., 2019). For example, the dataset of the Snapshot Serengeti (SS) Savannah Ecological Monitoring Project in Serengeti, Tanzania, Africa, collected 3.2 million images, of which 75% were empty images (Swanson et al., 2015). Automatically identifying and removing empty images from massive image datasets can significantly reduce the workload of ecologists (Harris et al., 2010; Hines et al., 2015; Rovero et al., 2013).

In recent years, deep learning (LeCun et al., 2015), a machine‐learning algorithm, has shown excellent performance in image processing and speech recognition (Simonyan & Zisserman, 2014; Verma & Gupta, 2018; Zhang et al., 2016). It enables the computer to extract features hierarchy from the raw data (images) and map the inputs (images) to some specific outputs (classes). The Deep Convolutional Neural Networks (DCNN) is one of the most popular deep learning algorithms and is widely used in image classification. Empty image recognition and species recognition based on DCNN have become a research hotspot in computer vision and machine learning (Beery et al., 2018; Giraldo‐Zuluaga et al., 2017; Gomez et al., 2017; Willi et al., 2019).

Generally, building a DCNN model requires a large number of labeled training samples, and the more training samples, the better the model tends to perform. Existing research uses millions of large‐scale training samples to build DCNN models to identify empty camera images (Norouzzadeh et al., 2018; Tab ak et al., 2019; Willi et al., 2019; Yousif et al., 2019). They have achieved high accuracy and small omission error that refer to the possibility that animal images are incorrectly predicted as empty images. However, manual labeling a large‐scale training set requires a lot of time and personnel costs. Many small ecological monitoring projects do not have the ability to label large‐scale training images. Reducing the number of training samples can save costs substantially, but the model usually produces a large omission error when the training sample size is small. For example, Willi et al. (2019) used datasets contained ~0.5 million images and the ResNet‐18 model (He et al., 2016) to identify empty images. They achieve high accuracy, but their omission error and commission error are 11.9% (100%‐88.1%) and 12.9% (100%‐87.1%) (see Figure 9 of Willi et al. (2019)), respectively. Furthermore, they indicated “that their method was more likely to miss an animal rather than detecting one that is not present.” The main purpose of ecological monitoring is to collect species information as much as possible. A large omission error of animal images may lead to missing the opportunities to discover species and observe wildlife. When the dataset is small, how to build the deep learning model to limit the omission error to an acceptable range and to maximize the automatic removal rate of empty images is still a challenge.

Ensemble learning (Breiman, 1996; Galar et al., 2012; Huynh et al., 2016) can combine different DCNN models to obtain better performance than any individual DCNN model using proper strategies. It has been well studied in remote sensing image processing and other aspects (Chen et al., 2019; Hurt et al., 2019;; Pathak et al., 2018; Xia et al., 2018) and proven to improve model performance. However, the research on the automatic recognition of empty camera trap images based on ensemble learning has just begun (Norouzzadeh et al. 2018). Norouzzadeh et al., (2018) use a large training set (1.4 million) and nine DCNN models to build an ensemble learning model to identify empty images automatically, but the accuracy of their ensemble model is not higher than that of the individual model.

Although the existing research using ensemble learning methods to recognize empty images does not significantly improve the recognition accuracy, it is still worth trying when only a small labeled dataset is available. Different from existing research, we pay more attention to the accuracy of empty image recognition, hoping to remove animal images as little as possible when automatically removing empty images. Therefore, we adopt a series of conservative strategies for the automatic removal of empty images. Here, the conservative strategies mean that we would rather identify empty images as animal images than identify animal images as empty images. According to this idea, we proposed an ensemble learning approach based on small‐scale dataset to remove empty camera trap images automatically. The goals of this study were:

1. To build the ensemble model based on a small data set to minimize omission error of animal images and avoid losing too much species information due to the automatic identifying and removal of empty images.
2. To maximize the proportion of empty images automatically removed by the model to reduce the burden of manually identifying and removing empty images.
3. To provide different empty images automatically identifying and removing schemes for users who have different acceptances of omission errors.

## 2 DATASETS AND METHODS

### 2.1 Dataset

Our study data came from 135 camera sites in Lhasa Mountain (LSM) (26°20′N, 99°15′E), Yunling Nature Reserve, Yunnan, China. We gathered all images of the 135 sites from May 2017 to August 2018. Whenever movement objects, such as waving vegetation, moving animals, etc., trigger the camera trap, the camera will take a set of images. Each trigger is called a capture event, and each capture event usually contains three images. A few capture events contain less than three images due to image corruption and other reasons. The LSM dataset contained 90,182 capture events (268,484 images) of 40 different species. Each image in the LSM dataset was assigned an empty or species label using our software system, called "Camera Trap Images Manual Classification System" (Yang et al., 2019). All images with species labels were treated as nonempty images, also called animal images. In the LSM dataset, the proportion of empty images was 77.86%, all images were in RGB format, and their size was 4,000 × 3,000 pixels. The LSM dataset contained all images taken during the day and night. We resized all images to meet the requirements of DCNN models for the input image size using the resize() method in the Python Imaging Library (Clark, 2019) of the Python 3.5.3 platform.

### 2.2 Ensemble learning framework

**FIGURE 1:**
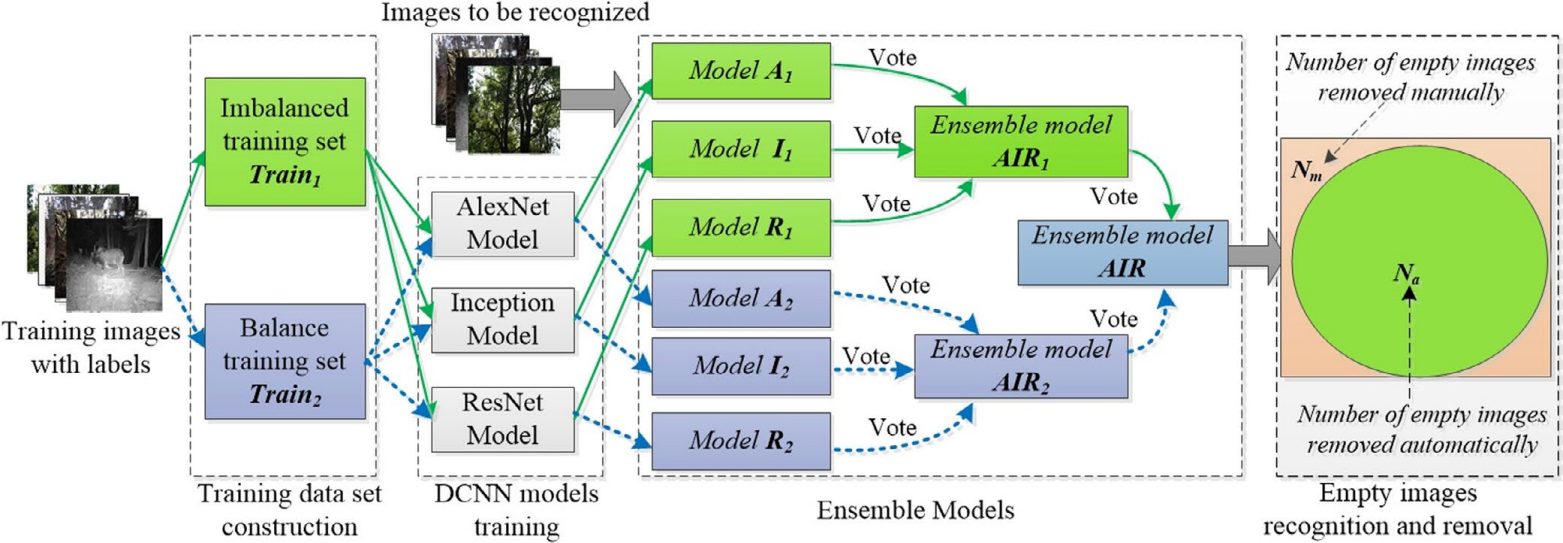
Framework of ensemble learning for automatically removing empty images. *A_i_, I_i_* and *R_i_* were the classifiers that output by the three DCNN models on the training set *Train_i_. AIR_i_* was the first‐level ensemble classifier combined with *A_i_, I_i_*, and *R_i_* (*i* = 1,2). *AIR* was the second‐level ensemble classifier combined with *AIR_1_* and *AIR_2_*

Based on the idea of conservatively removing empty images, we designed a multilevel ensemble learning framework (Figure 1). The goal of the ensemble learning framework was to maximize the *N_a_* and to minimize *N_m_* without losing species, where *N_a_* and *N_m_* were the numbers of empty images removed by the model automatically and by humans manually, respectively. To improve the model performance, we chose three DCNN models with significant differences in architecture as the basic model, then used a series of conservative strategies to combine these DCNN models. In consideration of the diversity of DCNN model architectures, our ensemble learning framework used three DCNN models: the AlexNet model (Krizhevsky et al., 2012), Inception model (Szegedy et al., 2016), and ResNet model (He et al., 2016). On the other hand, considering the impact of the class imbalance of the training dataset on the performance of the model, we constructed an unbalanced training set (*Train_1_*) and a balanced training set (*Train_2_*). We trained these three DCNN models on both training sets and then combined them, respectively. A common problem of DCNN‐based classifiers is that the number of samples of some classes in the training set is significantly higher than that of other classes, which is the called class imbalance problem (Chawla, 2010; Japkowicz & Stephen, 2002; Mazurowski et al., 2008). The performance of the DCNN model is susceptible to the class imbalance of the training set, and the classifier trends to recognize the input as the majority class in the training set (Buda et al., 2018). Combining models trained on different training sets using proper strategies can improve the performance of the model.

In the ensemble learning framework, we first trained the AlexNet, Inception, and ResNet models on the training set *Train_1_* to generate three models *A_1_, I_1_*, and *R_1_* and trained the models on training set *Train_2_* to generate models *A_2_, I_2_*, and *R_2_*. Second, we used a conservative strategy to combine *A_1_, I_1_,* and *R_1_* to obtain the first‐level ensemble model *AIR_1_* and to combine *A_2_, I_2_*, and *R_2_* to obtain the first‐level ensemble model *AIR_2_*. Finally, we used different conservative strategies to combine *AIR_1_* and *AIR_2_* to obtain the second‐level ensemble model *AIR*.

#### 2.2.1 Data splitting

In the camera trap image dataset, the images of the same capture event were overly similar. If images of the same capture event were split into the training set and the test set, the model often shows excellent performance on the test, but the generalization of the model was poor. To avoid this problem, we the same method as Norouzzadeh et al.,(2018) to put all the images of the entire capture event into either the training set or test set. We first randomly selected 80,000 capture events (238,673 images) from the LSM dataset to construct the training set *Train_1_*, which was an imbalanced training set. Then, we extracted all nonempty images in *Train_1_* and randomly selected the same number of empty images from *Train_1_* to construct the training set *Train_2_*, which was a balanced training set. Finally, we took the remaining 10,182 capture events (29,811 images) in the LSM dataset as the test set (*Test*). The description of the training and test sets was shown in Table 1.

**TABLE 1:** The training sets and the test set

Datasets	Number of total images	Number of empty images	Number of nonempty images	Empty image percentage (%)
*Train_1_*	238,673	185,688	52,985	77.80
*Train_2_*	105,970	52,985	52,985	50.00
*Test*	29,811	23,294	6,517	78.14

#### 2.2.2 DCNN models

We used the three DCNN models of AlexNet, InceptionV3, and ResNet‐18, because there are two core factors in defining a good ensemble classification system: the accuracy of individual classifiers and the diversity among classifiers (Faria et al., 2014). Since the AlexNet model was proposed in 2012, the depth and width of DCNN model have been increasing. The representative models of expanding the network width include NiN (Lin et al., 2013), GooleNet (Szegedy et al., 2015), Inception, etc. The representative models of expanding the network depth include VGG (Simonyan & Zisserman, 2014), ResNet, etc. The AlexNet model, winning the 2012 ImageNet Large‐scale Visual Recognition Challenge (ILSVRC 2012) challenge, is a landmark architecture for deep learning. It uses fewer levels and larger convolution kernels. The Inception model is proposed based on the GoogleNet model that won the 2014 ILSVRC challenge. The InceptionV3 model is the third version of Inception, which increases the width of the network and introduces the Batch Normalization algorithm and the idea of convolution kernel factorization. The ResNet model, winning the 2016 ILSVRC challenge, increases the network depth and introduces a residual module to solve the problem of network degradation. Experimental results of Norouzzadeh et al. (2018) showed that AlexNet, GoogleNet, and ResNet all achieve high accuracy for the task of empty and nonempty image recognition, and the accuracy of ResNet‐18 is 0.3% higher than that of ResNet‐152. Here ResNet‐18 is the simplified version of ResNet. The description of the three models was shown in Table 2.

**TABLE 2:** Characteristics of different deep learning architectures

Architecture	Number of layers	Input size	Short description
AlexNet	8	227 × 227	2012 ILSVRC Champion. It is a landmark architecture for deep learning.
InceptionV3	42	299 × 299	It increases the width of the network and uses the idea of Batch Normalization and factorization.
ResNet‐18	18	224 × 224	It introduces a residual module to solve the problem of network degradation.

#### 2.2.3 Confidence threshold and coverage

The outputs of the DCNN classifier are the probabilities that the input image belongs to all classes. The sum of these probabilities is 1. In other words, if we train a model to distinguish between empty and nonempty images, it will output the probabilities that the input image is empty and is an animal image, respectively. The probabilities can be interpreted as a confidence measure of the prediction result of the model. The class with the higher probability will be assigned to the input image as its label. Generally, the higher the prediction probability, the more credible the prediction result. Therefore, we can apply a confidence threshold to filter the prediction results of the model automatically. When the prediction probability output by the model is less than a certain threshold, it means that the model has little confidence in its prediction result, and the opinions of the model can be ignored. Ignoring low‐confidence predictions can improve the overall accuracy of the model, but it will also reduce its coverage. The coverage refers to the share of the model's prediction results that are accepted. For example, the model gives predictions of 100 images, of which prediction probabilities of 80 images exceed the confidence threshold, and the prediction probabilities of the other 20 images are below the confidence threshold. For this case, since we only accept 80 prediction results that exceeded the confidence threshold, the coverage is 80%.

#### 2.2.4 Ensemble methods

To achieve low omission error and meet the demands of users with different acceptances of omission error, we proposed a two‐level ensemble method. The first level was the DCNN model ensemble, which used a conservative strategy to combine DCNN models trained on the same training set into an ensemble model. The second level was the scheme ensemble, which used different conservative strategies to construct different empty image automatic removal schemes for users with different tolerances for omission errors.

In the DCNN model ensemble, we first set a 95% confidence threshold for each DCNN model. After setting the confidence threshold, the output of the DCNN model was one of the empty image, nonempty image, and uncertain image, which were represented as 0, 1, and δ, respectively. The uncertain images represented that the model's predicted probability for the input image was lower than the preset threshold. Then, we adopted a conservative strategy to combine DCNN model *A_1_, I_1_*, and *R_1_* into the ensemble model *AIR_1_* and to combine DCNN model *A_2_*, *I_2_*, and *R_2_* into the ensemble model *AIR_2_*. In our conservative strategy, the ensemble model *AIR_i_* (*i* = 1,2) assigned an empty (or nonempty) label to the input image if the predictions of model *A_i_*, *I_i_*, and *R_i_* for the same input image were all empty (or nonempty). Otherwise, the ensemble model *AIR_i_* assigned an uncertain label *x* to the input image. The reason why the ensemble model *AIR_i_* assigned an uncertain label *x* to the input image was that the prediction results of the three DCNN models for the same input image were inconsistent or were less than the threshold δ. Images with uncertain labels need to be identified and removed manually. We adopted such a conservative ensemble strategy because we hoped to reduce the probability of erroneously predicting nonempty images as empty images as much as possible. The conservative DCNN model ensemble method was defined as Equation (1).

(1) $$AIR_{i}=\left\{\begin{array}{cc} 0, & if\;A_{i}=I_{i}=R_{i}=0\\ 1, & if\;A_{i}=I_{i}=R_{i}=1\\ x, & \mathrm{else} \end{array}\right.,\;i=1,2$$

In the scheme ensemble, to meet the needs of different users for automatically removing empty images, we designed an enhanced ensemble strategy and a complementary ensemble strategy (Table 3). The purpose of the enhanced ensemble strategy was to achieve smaller omission errors to provide automatically removing services of empty images for users with a strict limitation for omission error. In the enhanced ensemble strategy, if both the ensemble model *AIR_1_* and the *AIR_2_* output the same prediction label for the same input image, the ensemble model *AIR* (denoted as *AIR_1_ and AIR_2_*) assigned the label to the input image. Otherwise, the ensemble model *AIR* assigned an uncertain label *x* to the input image. The purpose of the complementary ensemble strategy was to increase the coverage of the ensemble model *AIR* (denoted as *AIR_1_ or AIR_2_*) so that it can automatically remove more empty images while limiting the omission errors to an acceptable range. The complementary ensemble strategy was described as follows: (1) If the model *AIR_1_* and *AIR_2_* output the same prediction label for the same input image, the label was assigned to the input image; (2) if only one of the model *AIR_1_* and *AIR_2_* assigned a certainty (empty or nonempty) label to the input image, and the other assigned an uncertain label *x* to the input image, then the *AIR* assigned the certainty (empty or nonempty) label to the input image; and (3) if models AIR1 and AIR2 output certainty but opposite labels, then AIR assigned the nonempty label to the input image. It was a conservative strategy, which can reduce the probability that the model recognized animal images as empty images and removed them by mistake.

**TABLE 3:** Two different ensemble methods^a^

Model	Predicted results								
*AIR_1_*	0	0	0	1	1	1	*x*	*x*	*x*
*AIR_2_*	0	1	*x*	0	1	*x*	0	1	*x*
*AIR_1_* and *AIR_2_*	0	*x*	*x*	*x*	1	*x*	*x*	*x*	*x*
*AIR_1_* or *AIR_2_*	0	*1*	0	*1*	1	1	0	1	*x*

^a^ 0, 1, and *x* represented empty image, nonempty, and uncertain image, respectively. *AIR_1_* and *AIR_2_* was the enhanced ensemble model. *AIR_1_* or *AIR_2_* was the complementary ensemble model.

### 2.3 Model evaluation

Existing research treated empty images and animal images equally. They usually used accuracy to evaluate model performance, and some researchers used the recall and precision to evaluate their model. Our purpose was to look for an approach to achieve a small omission error at the cost of a reduced removal rate of empty images when only a small training set was available. We hoped to maximize the number of empty images that can be automatically removed by the model while limiting the number of animal images that were removed improperly. Therefore, we were more concerned about how many empty images the model correctly removed and how many animal images have been removed mistakenly. So we focused on three metrics: the omission error of animal images (*E_oa_*), the commission error of empty images (*E_ce_*), and the removal rate of empty images (*R_re_*), which were defined as (2), (3), and (4), respectively. In addition, to systematically evaluate the performance of the model, we supplemented two metrics that the overall error (*E_ov_*) and the commission error of the animal image (*E_ca_*), which were defined as (5) and (6).

(2) $$E_{\mathrm{oa}}=FN/\left(FN+TP\right)$$

(3) $$E_{\mathrm{ce}}=FN/\left(FN+TN\right)$$

(4) $$R_{\mathrm{re}}=TN/N_{e}$$

(5) $$E_{\mathrm{ov}}=\left(FN+FP\right)/\left(TP+TN+FN+FP\right)$$

(6) $$E_{\mathrm{ca}}=FP/\left(TP+FP\right)$$

Here, *TP* was the true positive, which indicated the number of images that the ground truth labels were animal images and were predicted to be animal images by the model. *FP* was the false positive, which indicated the number of images that the ground truth labels were empty images but were predicted to be animal images by the model. *FN* was the false negative, which indicated the number of images that the ground truth labels were animal images but were predicted to be empty images by the model. *TN* was the true negative, which indicated the number of images that the ground truth labels were empty and were predicted to be empty by the model. *N_e_* was the number of empty images in the test set. The *E_oa_* was the same as 1‐recall = 1 − *TP* / (*FN*+TP), but the *E_oa_* can more intuitively reflect the proportion of animal images that were falsely predicted by the model to the total number of animal images in the test set. The *E_ce_* intuitively reflected the proportion of animal images in the empty images removed by the model automatically. *R_re_* reflected the saved workload of automatically removing empty images.

Inputting an image, the model will output the corresponding prediction result. Some of the existing researches gave the recognition result of individual images, called image‐level results. Some researchers gave the recognition result of capture events, called event‐level results. So we gave both experimental results of the image‐level and the capture event‐level in this work.

## 3 RESULTS

### 3.1 Image‐level results

**FIGURE 2:**
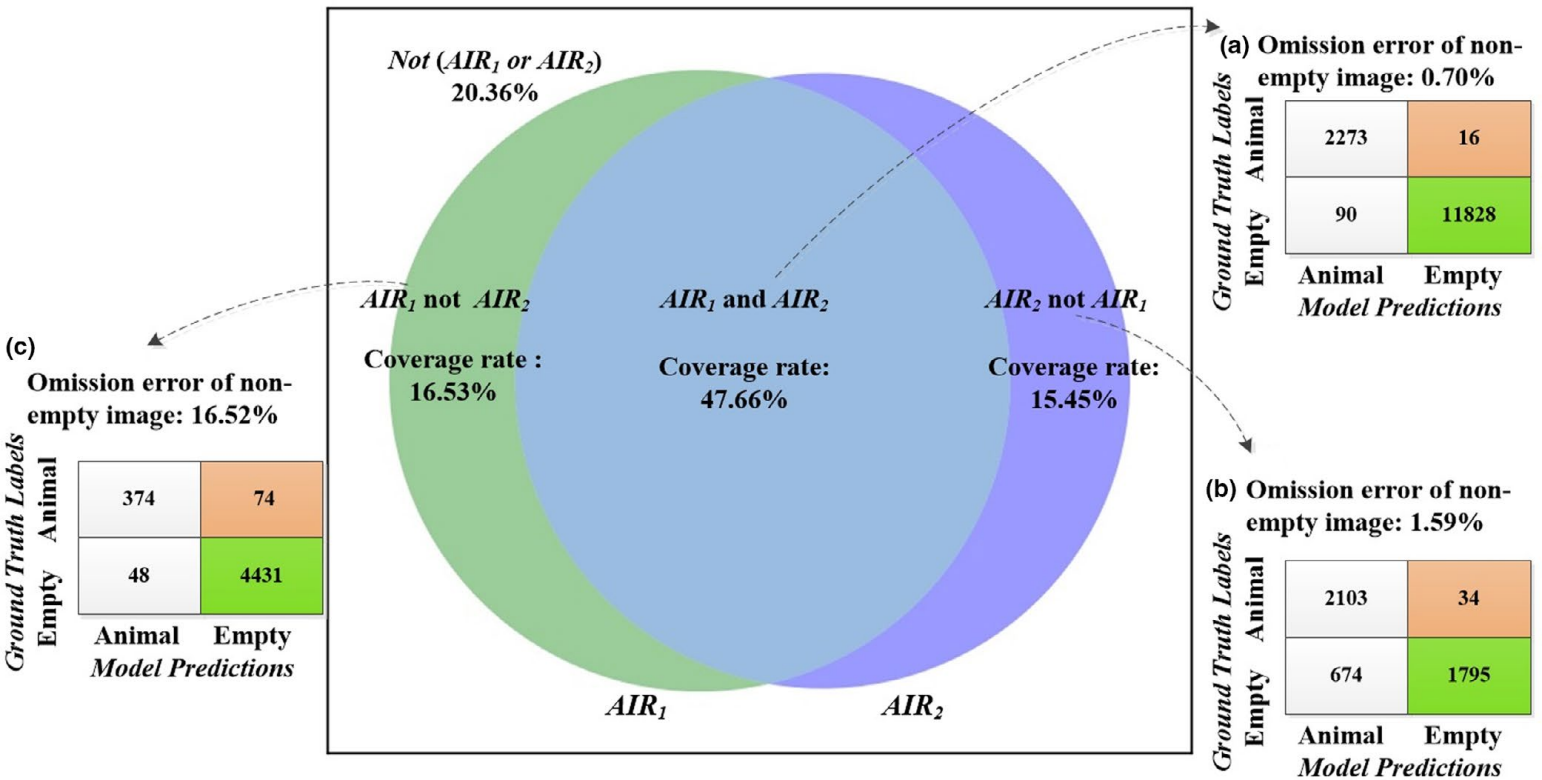
Image‐level experimental results on LSM dataset (with 95% confidence of DCNN models)

For the image‐level results, the ensemble model assigned a label to each input image, and we analyzed the experimental results based on the individual image (Figure 2). The coverage of the enhanced ensemble model *AIR* (i.e., *AIR_1_ and AIR_2_*) was 47.66%, and its *E_oa_* was 0.70%. That was, the ensemble model *AIR* automatically processed 47.66% of images in the dataset and mistakenly removed 16 nonempty images (Figure 2a). The coverage of the complementary ensemble model *AIR* (i.e., *AIR_1_ or AIR_2_*) increased to 79.64%. Among them, 47.66% of images can be recognized by both model *AIR_1_* and model *AIR_2_*. The 15.45% of images can be recognized by model *AIR_2_* but not by model *AIR_1_*, which was denoted as *AIR_2_ not AIR_1_* (Figure 2b). The other 16.53% of images can be recognized by model *AIR_1_* but cannot by model *AIR_2_*, which was denoted as *AIR_1_ not AIR_2_* (Figure 2c).

Based on the experiment results, we provided three different schemes of empty images automatic identifying and removal for users with different acceptances of *E_oa_* (Table 4) (See the Supporting Information for the confusion matrix of models), which can automatically remove different proportions of empty images. In the case of the LSM dataset, the percentages of empty images that were automatically removed by three schemes were 50.78%, 58.48%, and 77.51% when the omission errors were 0.70%, 1.13%, and 2.54%, respectively. Users can choose an empty image automatic removal scheme according to their acceptance of the *E_oa_*. Although the *E_oa_* and *R_re_* of the three schemes gradually increase, the maximal *E_oa_* of them was also smaller than any individual DCNN model (Table 4). Images that cannot be automatically identified by the three schemes need to be identified and removed manually. In the case of the LSM dataset, the proportions of empty images that need to be identified and removed manually in the three schemes were 49.22%, 41.52%, and 22.49%, respectively.

**TABLE 4:** Image‐level errors on the LSM dataset^a^

No.	Models	*E_ov_ (%)*	*E_oa_ (%)*	*E_ca_ (%)*	*E_ce_ (%)*	*R_re_ (%)*
I	*AIR_1_ and AIR_2_*	0.75	0.70	3.81	0.14	50.78
II	*AIR_2_*	4.33	1.13	14.86	0.37	58.48
III	*AIR_1_ or AIR_2_*	3.94	2.54	14.60	0.68	77.51
1	*AlexNet_1_*	8.10	8.8	23.58	2.59	86.89
2	*Inception_1_*	3.26	9.06	9.24	1.96	86.21
3	*ResNet‐18_1_*	9.82	22.18	26.28	5.51	80.61
4	*AlexNet_2_*	10.95	5.43	31.56	1.75	81.29
5	*Inception_2_*	6.80	3.80	22.31	1.12	85.14
6	*ResNet‐18_2_*	15.62	8.85	40.07	3.00	66.56

^a^ *E_ov_* was the overall error, and *E_ov_* = (*FP* + *FN*)/(*TP* + *FP* + *FN* + *TN*). *E_oa_* was the omission error of animal images, *and E_oa_* = 1‐*recall* = *FN* / (*FN* + TP). *E_ca_* was commission error of animal image, and *E_ca_ =1‐precision=FP/(TP* + *FP)*. *E_ce_* was the commission error of empty images, and *E_ce_* = *FN* / (*FN* + *TN*). *R_re_* was the removal rate of empty images, and *R_re_* = *TN* / *N_e_*, where *N_e_* was the number of empty images in the test set.

#### 3.1.1 Event‐level results

For the event‐level recognition results, the model still assigned a class label to each input image, but we analyzed the experimental results based on the captured events. To evaluate the experimental results of the capture event‐level, we defined empty capture events and nonempty capture events. That was, a capture event was an empty event if all images in the capture event were empty images, and a capture event was a nonempty (animal) event if at least one image in the capture event was an animal image. Our test set contained 10,182 capture events, of which the empty event rate was 75.99% (7,737 empty capture events).

**FIGURE 3:**
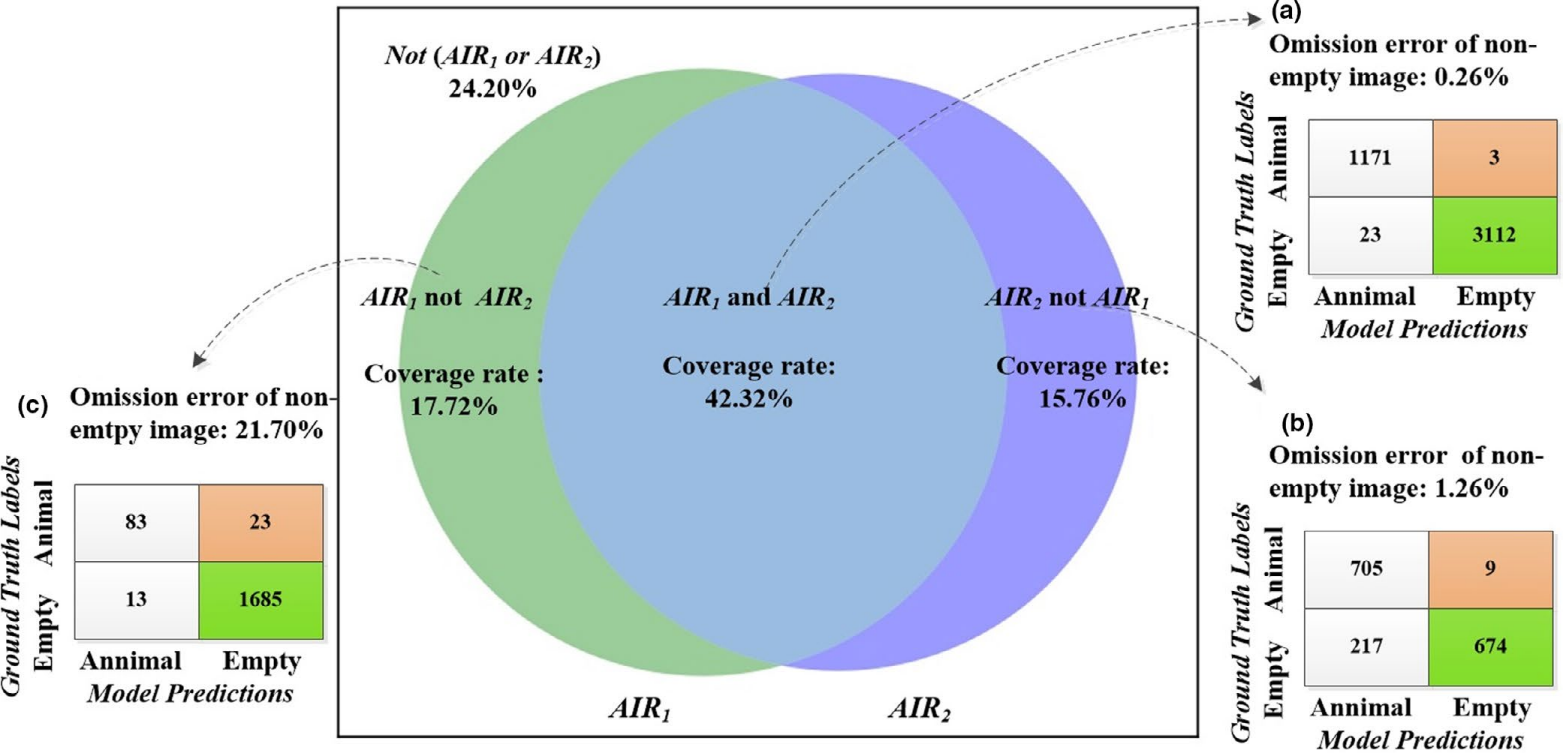
Event‐level experimental results on LSM dataset (with 95% confidence of DCNN models)

The coverage of the enhanced ensemble model *AIR* (i.e., *AIR_1_* and *AIR_2_*) was 42.32%, and the omission error of animal events was 0.26%. That was, the ensemble model *AIR* automatically processed 42.32% of the capture events in the dataset while mistakenly removing 3 animal events (Figure 3a). The coverage of the complementary ensemble model *AIR* (i.e., *AIR_1_* or *AIR_2_*) increased to 75.80%. Among them, 42.32% of events can be recognized by both models *AIR_1_* and mode *AIR_2_*. The 15.76% of events can be recognized by the model *AIR_2_* but not by the model *AIR_1_* (Figure 3b). The other 17.72% of events can be recognized by the model *AIR_1_* but not by the model *AIR_2_* (Figure 3c).

We also evaluated the event‐level results of these three schemes and individual model (Table 5). When the omission errors of animal events were 0.26%, 0.64%, and 1.61%, the three schemes automatically removed 40.22%, 48.93%, and 70.71% of empty events, respectively. The percentages of empty capture events that need to be identified and removed manually in three schemes were 59.78%, 51.07%, and 29.29%, respectively.

**TABLE 5:** Event‐level errors on the LSM dataset^a^

No.	Models	*E_ov_ (%)*	*E_oa_ (%)*	*E_ca_ (%)*	*E_ce_ (%)*	*R_re_ (%)*
I	*AIR_1_ and AIR_2_*	0.60	0.26	1.93	0.10	40.22
II	*AIR_2_*	4.26	0.64	11.34	0.32	48.93
III	*AIR_1_ or AIR_2_*	3.69	1.61	10.91	0.58	70.71
1	*AlexNet_1_*	8.25	7.12	22.26	2.46	84.99
2	*Inception_1_*	2.91	6.11	7.71	1.62	85.55
3	*ResNet‐18_1_*	10.59	11.31	26.80	3.87	72.59
4	*AlexNet_2_*	10.68	4.53	28.54	1.70	79.77
5	*Inception_2_*	6.37	3.47	19.17	1.18	85.56
6	*ResNet‐18_2_*	19.85	4.03	41.93	1.97	56.47

^a^ *E_ov_* was the overall error, and *E_ov_* = (*FP* + *FN*)/(*TP* + *FP* + *FN* + *TN*). *E_oa_* was the omission error of animal images, *and E_oa_* = 1‐*recall*= FN / (*FN* + *TP*). *E_ca_* was commission error of animal image, and *E_ca_ =1‐precision=FP/(TP* + *FP)*. *E_ce_* was the commission error of empty images, and *E_ce_* = FN / (*FN* + *TN*). *R_re_* was the removal rate of empty images, and *R_re_* = *TN* / *N_e_*, where *N_e_* was the number of empty images in the test set.

## 4 DISSCUSION

### 4.1 Generalizability

To verify the generalizability of our ensemble learning approach based on the conservative strategies, we applied it to 332,192 images from 135 cameras of the SS benchmarking data set, which were extracted from S1 subset of the SS dataset and denoted as the SS_S1_135 dataset (see Supporting Information for more detail). The sample size of the SS_S1_135 dataset was equivalent to that of the LSM dataset. We built the training set and test set using the same method (Table S.7), and retrained and tested the models on the SS_S1_135. Experimental results showed that the ensemble models also performed better than any individual DCNN model on the SS_S1_135 dataset (Table 6). It can be seen that our ensemble learning ideas based on conservative strategies can be applied to the small dataset of the savanna ecosystem.

**TABLE 6:** Image‐level errors on the SS_S1_135 dataset (with 95% of confidence)^a^

No.	Models	*E_ov_ (%)*	*E_oa_ (%)*	*E_ca_ (%)*	*E_ce_ (%)*	*R_re_ (%)*
I	*AIR_1_ and AIR_2_*	0.71	2.75	2.69	0.42	86.29
II	*AIR_2_*	2.07	2.95	9.45	0.56	87.24
III	*AIR_1_ or AIR_2_*	2.43	5.68	9.45	1.06	94.02
1	*AlexNet_1_*	3.76	11.11	11.38	2.23	96.03
2	*Inception_1_*	2.74	11.35	5.71	2.20	97.08
3	*ResNet‐18_1_*	3.94	17.31	8.95	3.12	96.05
4	*AlexNet_2_*	5.41	7.26	20.73	1.56	93.27
5	*Inception_2_*	4.26	6.14	16.44	1.32	92.67
6	*ResNet‐18_2_*	5.07	8.14	19.79	1.65	91.06

^a^ *E_ov_* was the overall error, and *E_ov_* = (*FN*+ *FP*)/(*TP* + *FP* + *FN*+ *TN*). *E_oa_* was the omission error of animal images, *and E_oa_* = 1‐*recall*= *N* / (*FN* + *TP*). *E_ca_* was commission error of animal image, and *E_ca_ = 1‐precision= FP/(TP + FP)*. *E_ce_* was the commission error of empty images, and *E_ce_* = FN / (*FN* + *TN*). *R_re_* was the removal rate of empty images, and *R_re_* = *TN* / *N_e_*, where *N_e_* was the number of empty images in the test set.

Although the ensemble models obtained smaller errors than any individual model on two different datasets, there was still a significant difference between the model performances on the two different datasets. For example, the *E_oa_* of the ensemble model on the LSM data was smaller, and the *E_ca_* of the ensemble model on the SS_S1_135 dataset was smaller. These differences exist because the two datasets came from different ecosystems, with various natural environments and wildlife communities. LSM is a typical mountainous terrain dominated by primary forests, where harbors wildlife species with relatively small body size. The SS dataset is a typical savanna ecosystem, and most wildlife species trapped by cameras are large mammals.

### 4.2 Ensemble model versus individual model

The goal of this study was to identify and remove empty camera trap images accurately. Therefore, we preferred that the model achieved a smaller omission error. If a model has a high empty image removal rate, but its omission error is also high, such a model cannot be used in actual ecological projects. In this work, we balanced the removal rate of empty images with the omission error of animal images to limit the omission error to an acceptable range and remove empty images as many as possible automatically. In this perspective, our ensemble learning method performed better than the individual DCNN model on both data sets.

We found that in the first‐level ensemble, the models with the smallest omission errors on the balanced training set and the unbalanced training set were the Inception and the AlexNet, respectively. The *E_ov_* of the Inception model was always the best on both datasets. The three models showed consistency on the two sets of data.

Norouzzadeh et al. (2018) indicate that the ResNet‐18 model performed better than the AlexNet model. However, in most cases in our experiments, the ResNet‐18 model did not perform better than the AlexNet model on both LSM and SS_S1_135 datasets, especially on the LSM dataset. The possible reason was that our training samples (<0.24 million) are much less than that of Norouzzadeh et al. (2018) (1.4 million). We speculated that the ResNet‐18 required more training samples than AlexNet to achieve good accuracy, which will be further verified in our future work.

The AlexNet, Inception, and ResNet‐18 models were used as the basic models because the architectures of these three models were significantly different, and they all showed excellent accuracy in identifying the camera trap images (Norouzzadeh et al., 2018). They represent the starting point of the DCNN model and two improving directions, respectively. In this work, we focused on whether our ensemble models can achieve better performance on small‐scale datasets than the individual model. However, these three models were not necessarily the best combination. Regarding which models are the best combination, we will further explore them and then develop it into software for users for free downloading in future work.

### 4.3 The impact of different schemes on the frequency of species occurrence

The purpose of this study was to automatically identify and remove empty images as many as possible while keeping the *E_oa_* to a small range instead of automatically removing all the empty images in the dataset. For empty images that the models cannot automatically recognize, we recommended manual identifying and removing them. In this study, we assumed the manual recognition of empty images was credible. Based on this assumption and LSM dataset, we analyzed the impact of the omission errors of three empty image automatic removal schemes on the frequency of species occurrence. The scheme I missed 16 animal images, which were distributed in 12 capture events of 9 cameras (see Figure S12 in Supporting Information for the image examples). Among them, three capture events (8 images) were completely missed, and the remaining events only missed part of the images. Our scheme II missed 50 animal images, which were distributed in 29 capture events of 24 cameras, of which 12 capture events (27 images) were completely missed. Our scheme III missed 124 animal images, which were distributed in 76 capture events of 48 cameras, of which 32 capture events (84 images) were completely missed. We manually checked the 124 missed animal images and found that only 17.75% (22 images) of them can identify the species information, and the remaining 82.25% (102 images) can only find the presence of animals in the images, but it is hard to identify the species information. It means 82.25% of the nonempty images that were removed mistakenly were insignificance for ecological research. Our experimental results showed that although these three schemes for automatically removing empty images slightly changed the frequency of species occurrence, they did not miss the species information (see Figure S9 in Supporting Information for the detailed information).

### 4.4 Confidence threshold

**FIGURE 4:**
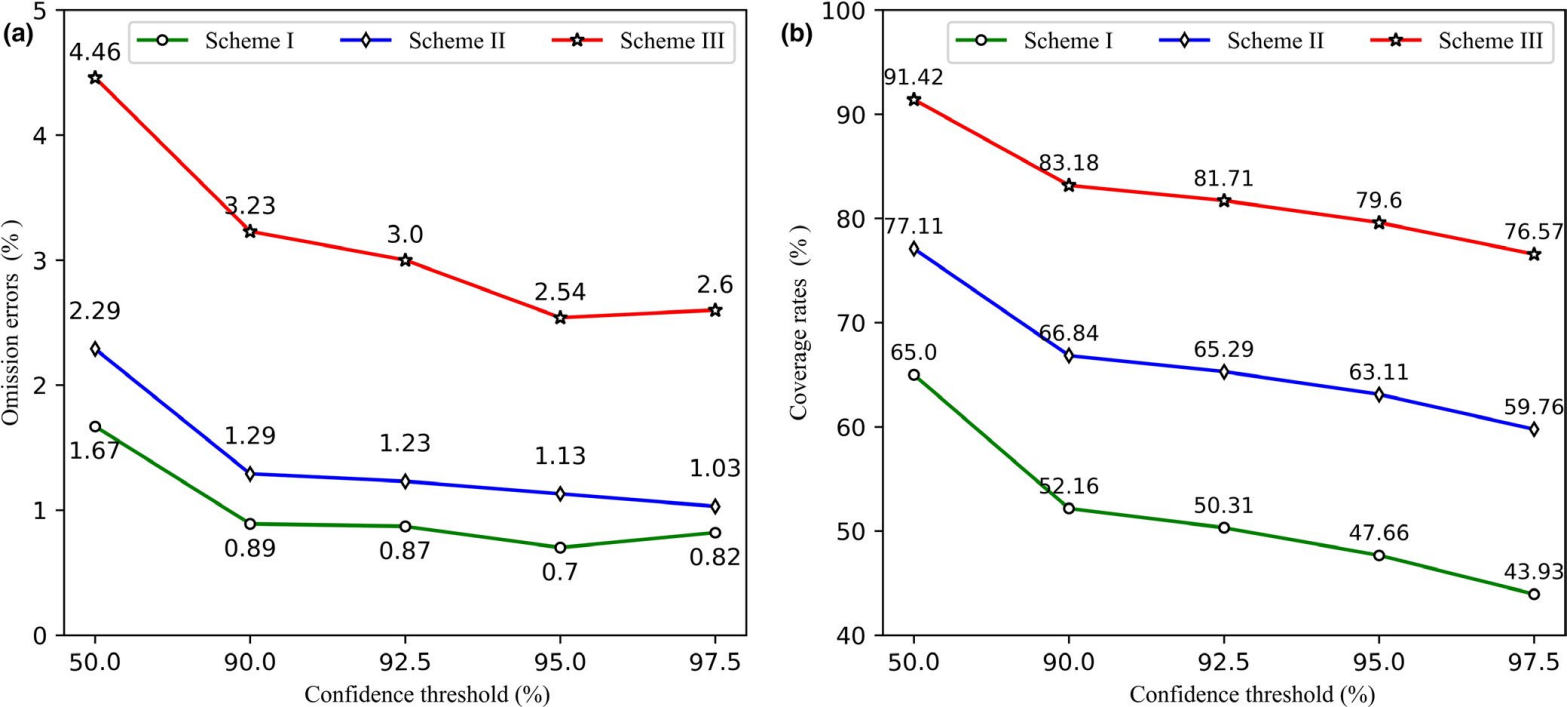
Omission errors of animal images (a) and coverage (b) of different schemes with different confidence thresholds on the LSM dataset

Setting a confidence threshold for the outputs of the DCNN models can significantly reduce the errors of the DCNN models. To study the impact of the confidence threshold of the DCNN model on the ensemble model performance, we tested the *E_oa_* and coverage of each scheme under different confidence thresholds (Figure 4) (see Table S3–S6 and Figure S6–S8 in Supporting Information for the more detailed results). We found that when the confidence threshold of DCNN models was 95%, the image‐level omission errors of the three schemes were 0.7%, 1.1%, and 2.5%, respectively, which represented different error gradients and provided different alternatives to users with different error tolerance. When the confidence threshold of the DCNN model was 97.25%, although the *E_oa_* of scheme II was still decreasing, the *E_oa_* of schemes I and III had begun to increase. We also found that when the confidence threshold of the DCNN model was not less than 90%, using any of our three schemes to automatically remove empty images did not miss the species information. However, if the confidence threshold of DCNN models was 50% (i.e., without confidence threshold), neither the scheme I nor the scheme II missed species information, but the scheme III (with 4.46% of *E_oa_*) missed a rare species (*Pomatorhinus ruficollis*). This missed species only appeared twice in the LSM dataset (two capture events), one event was divided into the training set, and the other was divided into the test set (see Figure S13 in Supporting Information for the missed species examples).

### 4.5 Limitations and future work

We found that no matter which empty image automatic filtering schemes (scheme *Ⅰ*, scheme *Ⅱ*, or scheme *Ⅲ*) was used, there were always some empty images that need to be manually identified and removed, which was the limitations of our method. For our three schemes (with a 95% confidence threshold of DCNN models), the percentages of images that we need to recognize manually were 52.34%, 36.89%, and 20.36%, respectively. In our future research, we will focus on improving the coverage of schemes while limiting the omission errors.

We attempted to use the majority rule to ensemble the six DCNN models (without the confidence threshold) to identify the 20.36% (6,701 images) of images, which cannot be processed automatically by scheme III and was denoted as *Not* *(AIR_1_ or AIR_2_)*. We did not set the confidence threshold of the six DCNN models so that each DCNN model can cover all test samples. For this attempt, the *E_oa_* was 14.73% (see Figure S14 in the Supporting Information for the confusion matrix). Furthermore, for users who were unwilling to remove empty images manually, we suggest the scheme IV. The scheme IV included two steps: (1) first, use scheme III to identify and filter empty images in the dataset automatically and (2), second, use the majority principle to ensemble the six DCNN models (without confidence) to identify and remove empty images that cannot be identified by scheme III. The *E_oa_*, *E_ce_*, and *R_re_* of scheme IV were 5.62%, 1.73%, and 89.50%, respectively (see Figure S15 in the Supporting Information for the confusion matrix of scheme IV).

On a small dataset, another way for the DCNN model to obtain better performance is the transfer learning (Bengio et al., 2013; Donahue et al., 2014; Yosinski et al., 2014). Transfer learning first pretrains the model on a publicly large‐scale dataset, then uses a small target dataset to retrain the model to obtain better performance than training directly on the small dataset. Our ensemble learning method is not opposed to the transfer learning. The individual DCNN model can be pretrained on the SS dataset firstly then transferred to our LSM dataset. After that, use our ensemble learning method to improve the performance of the model. In this way, maybe we only need fewer training samples to achieve smaller errors and a larger empty image removal rate, which will be further researched in our future work.

## CONFLICT OF INTERESTS

The authors declare that there is no conflict of interests regarding the publication of this article.

## AUTHOR CONTRIBUTIONS

**Deng Qi Yang:** Methodology (equal); Project administration (lead); Writing‐original draft (lead); Writing‐review & editing (equal). **Kun Tan:** Data curation (lead); Investigation (equal); Resources (equal). **Zhipang Huang:** Data curation (equal); Resources (equal). **Xiao Wei Li:** Software (equal). **Ben Hui Chen:** Software (equal). **Guopeng Ren:** Conceptualization (equal); Methodology (supporting); Project administration (supporting); Writing‐review & editing (equal). **Wen Xiao:** Conceptualization (equal); Project administration (equal); Supervision (equal).

## Supporting information

Supplementary Material

## Data Availability

The model codes and additional meta‐data can be accessed on the figshare (https://doi.org/10.6084/m9.figshare.13003922). Camera trap images of SS dataset can be accessed on Dryad (http://dx.doi.org/10.5061/dryad.5pt92). Camera trap images of LSM dataset are not publicly available to protect endangered species from poaching but the specific requirements can be addressed directly to the corresponding author by interested third parties.
