# Supplementary material for: An automatic method for removing empty camera trap images using ensemble learning
Source: Ecol Evol. 2021 May 2;11(12):7591–601. doi: 10.1002/ece3.7591 (PMC8216933; doi:10.1002/ece3.7591)
Supplement: Supplementary file 1 — Supplementary Material [file ECE3-11-7591-s001.docx]

Supporting Information for

**An automatic method for removing empty camera trap images using ensemble learning**

Deng-Qi Yang^1,2,3,4#^, Kun Tan^2,3#^, Zhi-Pang Huang^2,3^, Xiao-Wei Li^1,4^, Ben-Hui Chen^1,4^, Guo-Peng Ren^2,3*^, Wen Xiao^2,3^

^1^Department of Mathematics and Computer Science, Dali University, Dali, Yunnan, China;

^2^Institute of Eastern-Himalaya Biodiversity Research, Dali University, Dali, China;

^3^Collaborative Innovation Center for the Biodiversity in the Three Parallel Rivers of China, Dali 671003, China;

^4^Data Security and Application Innovation Team, Dali University, Dali, Yunnan, China

^*^Corresponding author: rengp@eastern-himalaya.cn

1. **Image preprocessing**

The size of the original images in LSS dataset is 3000×4000, which is too large for current state-of-the-art deep neural networks owing to the increased computational costs of training and running DNNs on high-resolution images. The requirements of the AlexNet, Inception, and ResNet-18 models for the input image size are 227×227, 299×299, and 224×224 respectively. We give three examples of scaling images from 3000×4000 to 227×227 (Fig.S.1).


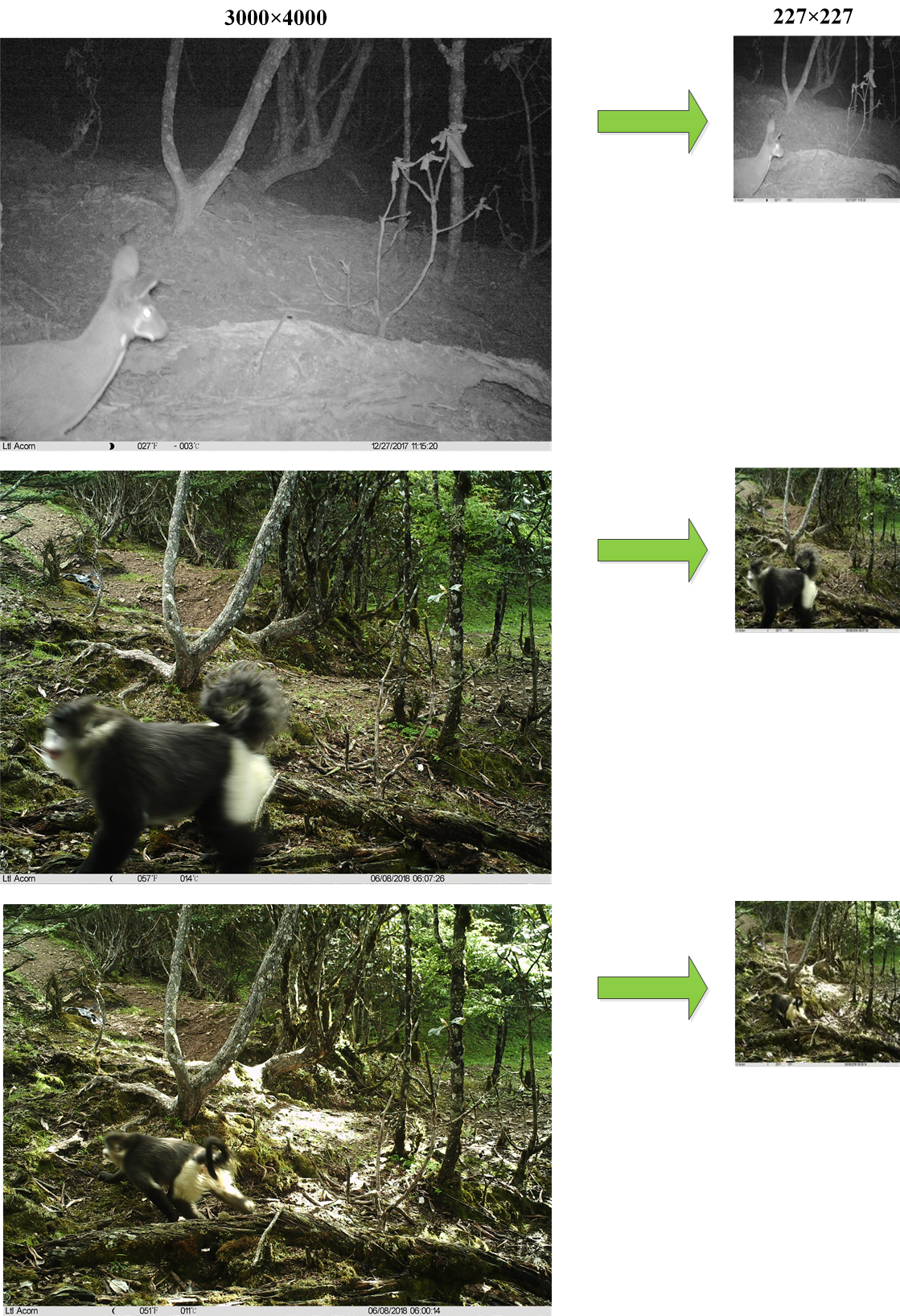


**Fig. S.1.** Examples of camera-trap images in the LSS dataset (left) and their downsampled images (right).

1. **Evaluation metrics**

We present some more detailed experimental results in this section. To compare with existing research, we used evaluation metrics in the Supporting Information: overall error (*E_oe_*), commission error of animal image (*E_ca_*), and the omission error of animal images (*E_oa_*), which are defined by equation (1) - (5).

*E_oe_ = ( FN+FP )/(TP+TN+FN+FP)* (1)

*E_oa_* = *FN* / (*FN*+*TP*) (2)

*E_ca_ =FP/(TP + FP)* (3)

*E_ce_* =*FN* / (*FN*+*TN*) (4)

*R_re_* = *TN* / *N_e_* (5)

Here, *TP* is the number of images that are labeled as animal images by both human and model. *TN* is the number of images that are labeled as empty images by both human and model. *FN* is the number of images that are labeled as animal images by human but labeled as empty images by the model. *FP* is the number of images that are labeled as empty by human but labeled as animals by the model.

1. **Additional result of the LSM dataset**

**3.1 The performance of the DCNN models under different confidence thresholds.**

3.1.1 Model confusion matrix of models with a 95% confidence threshold.


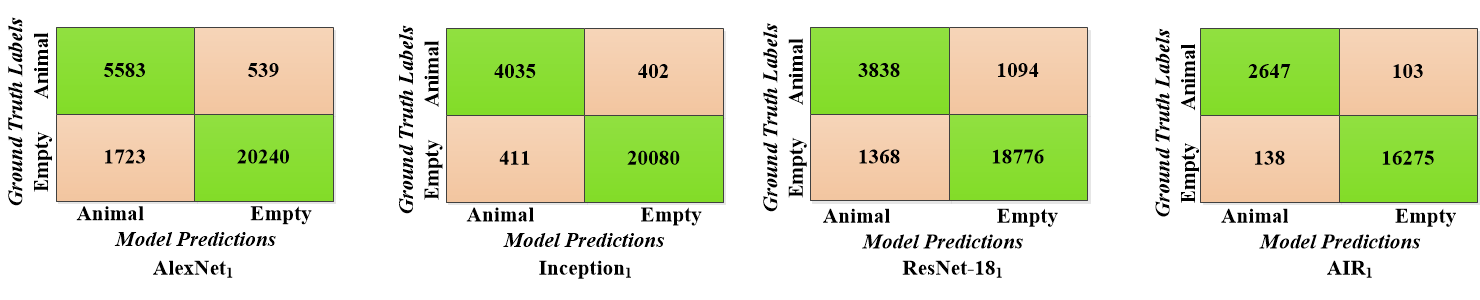


**Fig S.2** The confusion matrix of models on the unbalanced training set *Train_1_* (with a 95% confidence threshold).


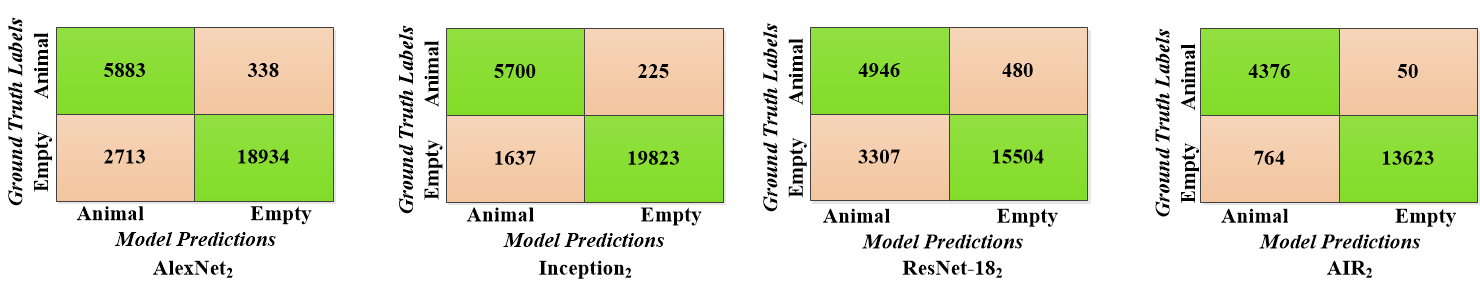


**Fig S.3** The confusion matrix of models on the unbalanced training set *Train_2_* (with a 95% confidence threshold).

3.1.2 Model performance without confidence threshold (i.e., confidence=50%).

- Table S.1 shows the performance of the individual DCNN models and the DCNN ensemble model *AIR_1_* (without the confidence threshold) using the imbalanced training set *Train_1_*. Figure S.4 shows the corresponding confusion matrixes of models.
- Table S.2 shows the performance of the individual DCNN models and the DCNN ensemble model *AIR_2_* (without the confidence threshold) using the imbalanced training set *Train_2_*. Figure S.5 shows the corresponding confusion matrixes of models.

**TABLE S.1** The image-level results of model on the unbalanced training set *Train_1_* (without the confidence threshold) ^*^

|  | ***E_ov_ (*%*)*** | ***E_oa_ (*%*)*** | ***E_ca_ (*%*)*** | ***E_ce_ (*%*)*** | ***R_re_ (*%*)*** |
| --- | --- | --- | --- | --- | --- |
| *AlexNet1* | 8.05 | 8.80 | 23.58 | 2.59 | 86.89 |
| *Inception_1_* | 3.26 | 9.06 | 9.24 | 1.96 | 86.21 |
| *ResNet_1_* | 9.82 | 22.18 | 26.28 | 5.51 | 80.61 |
| *Ensemble model (AIR_1_)* | 1.26 | 3.75 | 4.96 | 0.63 | 69.87 |

^*^ *E_ov_ was the overall error. E_oa_ was the omission error of animal images. E_ca_ was commission error of animal images. E_ce_ was the commission error of empty images. R_re_ was the removal rate of empty images.*


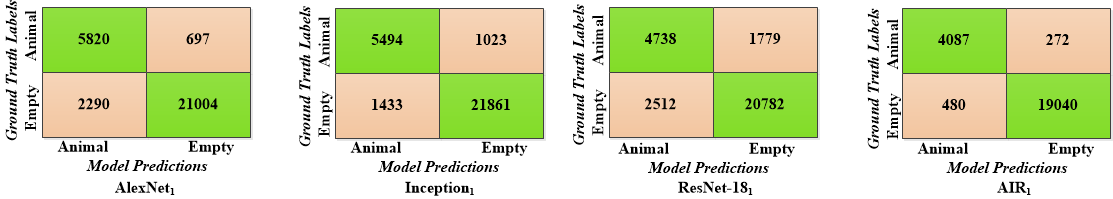


**Fig S.4** The confusion matrix of models on the unbalanced training set *Train_1_* (without the confidence threshold).

**TABLE S.2** The image-level results of model on the unbalanced training set *Train_2_* (without the confidence threshold) ^*^

|  | ***E_ov_ (*%*)*** | ***E_oa_ (*%*)*** | ***E_ca_ (*%*)*** | ***E_ce_ (*%*)*** | ***R_re_ (*%*)*** |
| --- | --- | --- | --- | --- | --- |
| *AlexNet1* | *13.03* | *7.17* | *36.10* | *2.30* | *85.33* |
| *Inception_1_* | *9.78* | *6.63* | *29.00* | *2.03* | *89.34* |
| *ResNet_1_* | *20.65* | *14.50* | *48.33* | *4.97* | *77.63* |
| *Ensemble model (AIR_1_)* | *6.45* | *2.18* | *20.92* | *0.70* | *70.12* |

^*^ *E_ov_ was the overall error. E_oa_ was the omission error of animal images. E_ca_ was commission error of animal images. E_ce_ was the commission error of empty images. R_re_ was the removal rate of empty images.*


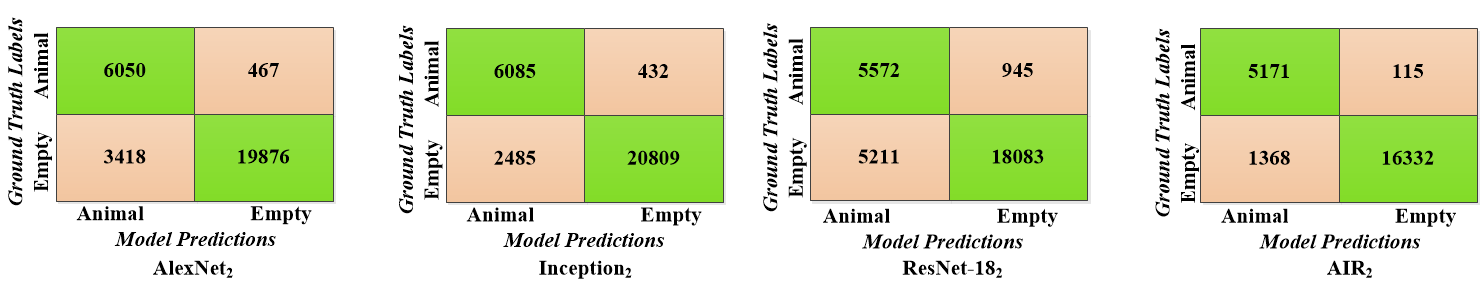


**Fig S.5** The confusion matrix of models on the unbalanced training set *Train_2_* (without the confidence threshold).

**3.2 The performance of different ensemble schemes with different confidence thresholds of DCNN models**

- Table S.3 shows the errors and coverage of the scheme *Ⅰ* under different confidence thresholds. Figure S.6 shows the corresponding confusion matrixes of models.
- Table S.4 shows the errors and coverage of the scheme *Ⅱ* under different confidence thresholds. Figure S.7 shows the corresponding confusion matrixes of models.
- Table S.5 shows the errors and coverage of the scheme *Ⅲ* under different confidence thresholds. Figure S.8 shows the corresponding confusion matrixes of models.
- Table S.6 shows the number of missed non-empty images of different schemes under different confidence thresholds.

**TABLE S.3** Errors and coverage rate of scheme *Ⅰ* under different confidence thresholds (%)^*^

|  | *E_ov_ (*%*)* | *E_oa_ (*%*)* | *E_ca_ (*%*)* | *E_ce_ (*%*)* | *R_re_ (*%*)* |
| --- | --- | --- | --- | --- | --- |
| *50.0* | 2.18 | 1.66 | 8.78 | 0.41 | 65.40 |
| *90.0* | 1.07 | 0.89 | 5.10 | 0.19 | 54.63 |
| *92.25* | 0.89 | 0.87 | 4.25 | 0.18 | 53.08 |
| *95.0* | 0.75 | 0.70 | 3.81 | 0.14 | 50.78 |
| *97.25* | 0.58 | 0.72 | 3.10 | 0.13 | 47.56 |

^*^ *E_ov_ was the overall error. E_oa_ was the omission error of animal images. E_ca_ was commission error of animal images. E_ce_ was the commission error of empty images. R_re_ was the removal rate of empty images.*


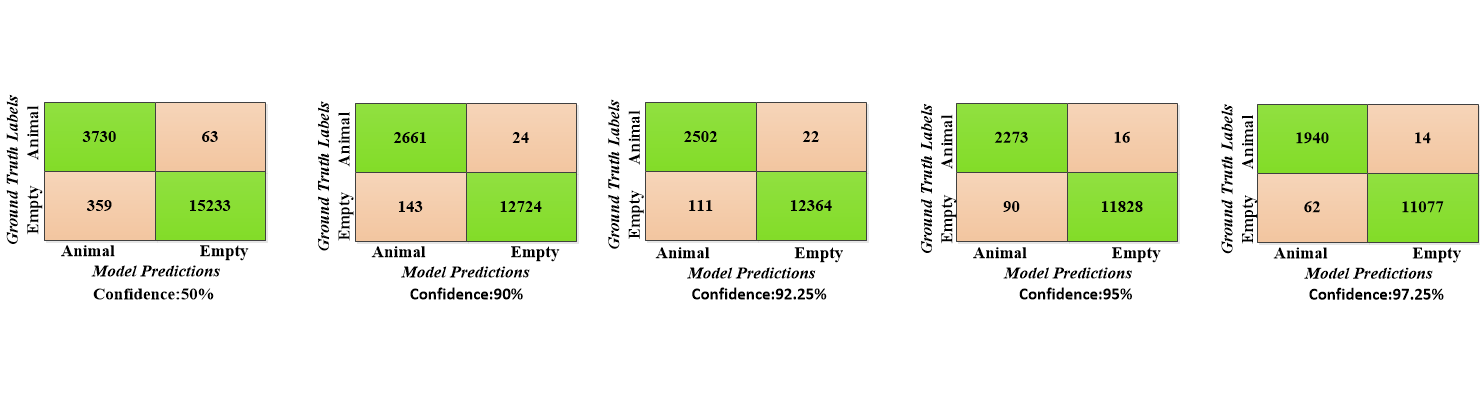


**Fig S.6** The confusion matrix of scheme *Ⅰ* under different confidence thresholds

**TABLE S.4** Error and coverage rate of scheme *Ⅱ* under different confidence thresholds (%)^*^

|  | ***E_ov_ (*%*)*** | ***E_oa_ (*%*)*** | ***E_ca_ (*%*)*** | ***E_ce_ (*%*)*** | ***R_re_ (*%*)*** |
| --- | --- | --- | --- | --- | --- |
| *50.0* | 6.45 | 2.18 | 20.92 | 0.70 | 70.12 |
| *90.0* | 4.82 | 1.29 | 16.34 | 0.42 | 61.64 |
| *92.25* | 4.65 | 1.22 | 15.84 | 0.40 | 60.28 |
| *95.0* | 4.33 | 1.13 | 14.86 | 0.37 | 58.49 |
| *97.25* | 3.90 | 0.96 | 13.64 | 0.31 | 55.72 |

^*^ *E_ov_ was the overall error. E_oa_ was the omission error of animal images. E_ca_ was commission error of animal images. E_ce_ was the commission error of empty images. R_re_ was the removal rate of empty images.*


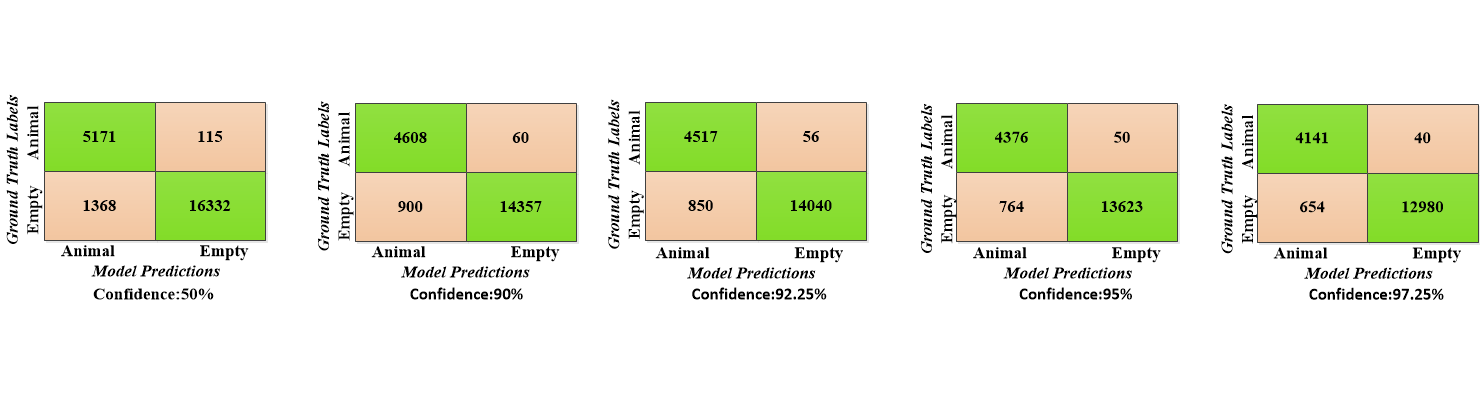


**Fig S.7** The confusion matrix of scheme *Ⅱ* under different confidence thresholds

**TABLE S.5** Error and coverage rate of scheme *Ⅲ* under different confidence thresholds (%)^*^

|  | ***E_ov_ (*%*)*** | ***E_oa_ (*%*)*** | ***E_ca_ (*%*)*** | ***E_ce_ (*%*)*** | ***R_re_ (*%*)*** |
| --- | --- | --- | --- | --- | --- |
| *50.0* | 6.41 | 4.46 | 21.16 | 1.28 | 85.68 |
| *90.0* | 4.53 | 3.23 | 16.05 | 0.89 | 80.14 |
| *92.25* | 4.33 | 2.99 | 15.58 | 0.81 | 78.99 |
| *95.0* | 3.94 | 2.54 | 14.60 | 0.68 | 77.51 |
| *97.25* | 3.56 | 2.59 | 13.32 | 0.68 | 75.15 |

^*^ *E_ov_ was the overall error. E_oa_ was the omission error of animal images. E_ca_ was commission error of animal images. E_ce_ was the commission error of empty images. R_re_ was the removal rate of empty images.*


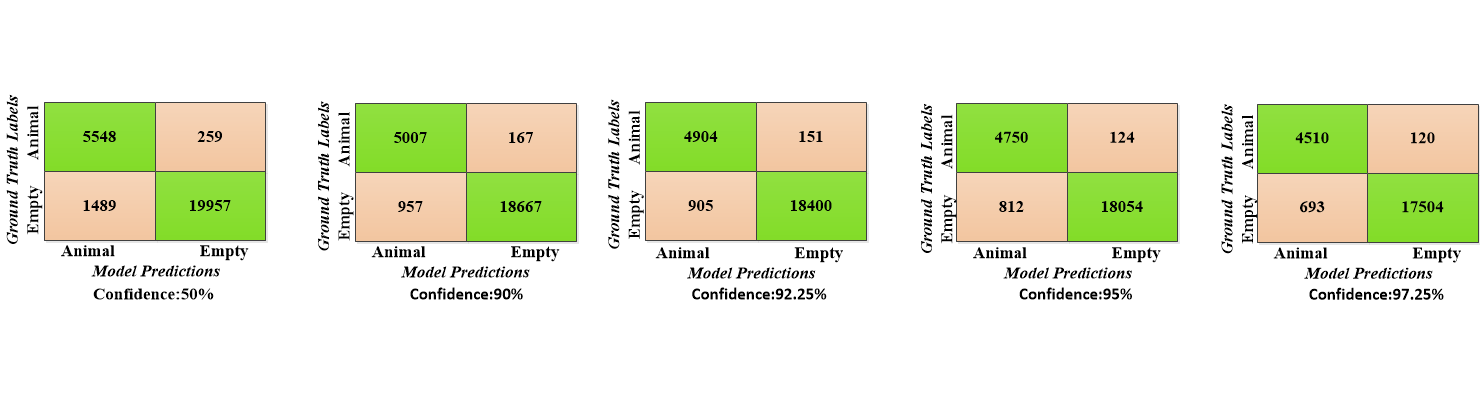


**Fig S.8** The confusion matrix of scheme *Ⅲ* under different confidence thresholds

**Table S.6** The number of animal images missed by different schemes under different confidence thresholds

|  | **50.0%** | **90.0%** | **92.5%** | **95.0%** | **97.5%** |
| --- | --- | --- | --- | --- | --- |
| Scheme *Ⅰ* | 63 | 24 | 22 | 16 | 16 |
| Scheme *Ⅱ* | 115 | 60 | 56 | 50 | 40 |
| Scheme *Ⅲ* | 259 | 167 | 151 | 124 | 120 |

**3.3 The species distribution and variation of species occurrence frequency in test set**

Figure S.9a-b show the species distribution and changes in occurrence frequency before and after automatic removal of empty images using scheme *Ⅰ* and scheme *Ⅲ*.

**Fig.S.9-a** The frequency of species occurrence and the number of missing animal images when using the schemes *Ⅰ* to automatically remove empty images.

**Fig.S.9-b** The frequency of species occurrence and the number of missing animal images when using the schemes *Ⅲ* to automatically remove empty images.

**3.4 The results of the ensemble models without the confidence threshold**

3.4.1 Image-level results

Figure S.10 shows the image-level results of the ensemble model without the confidence threshold. The enhanced ensemble model *AIR* (i.e., *AIR_1_ and AIR_2_*) that did not set the confidence threshold of DCNN models can automatically identify and filter 64.96% of images. The omission error of non-empty images (*E_oa_*), commission error of empty images (*E_ce_*), and removal rate of empty images (*R_re_*) were 1.61%, 0.41%, and 65.39%, respectively. The ensemble model mistakenly removed 63 non-empty images. The complementary ensemble model *AIR* (i.e., *AIR_1_* *or* *AIR_2_*) that did not set the confidence threshold of DCNN models can automatically identify and filter 91.40% of images. The *E_oa_*, *E_ce_*, and *R_re_* were 4.46%, 1.28%, and 85.67%, respectively. The ensemble model mistakenly removed 259 non-empty images.


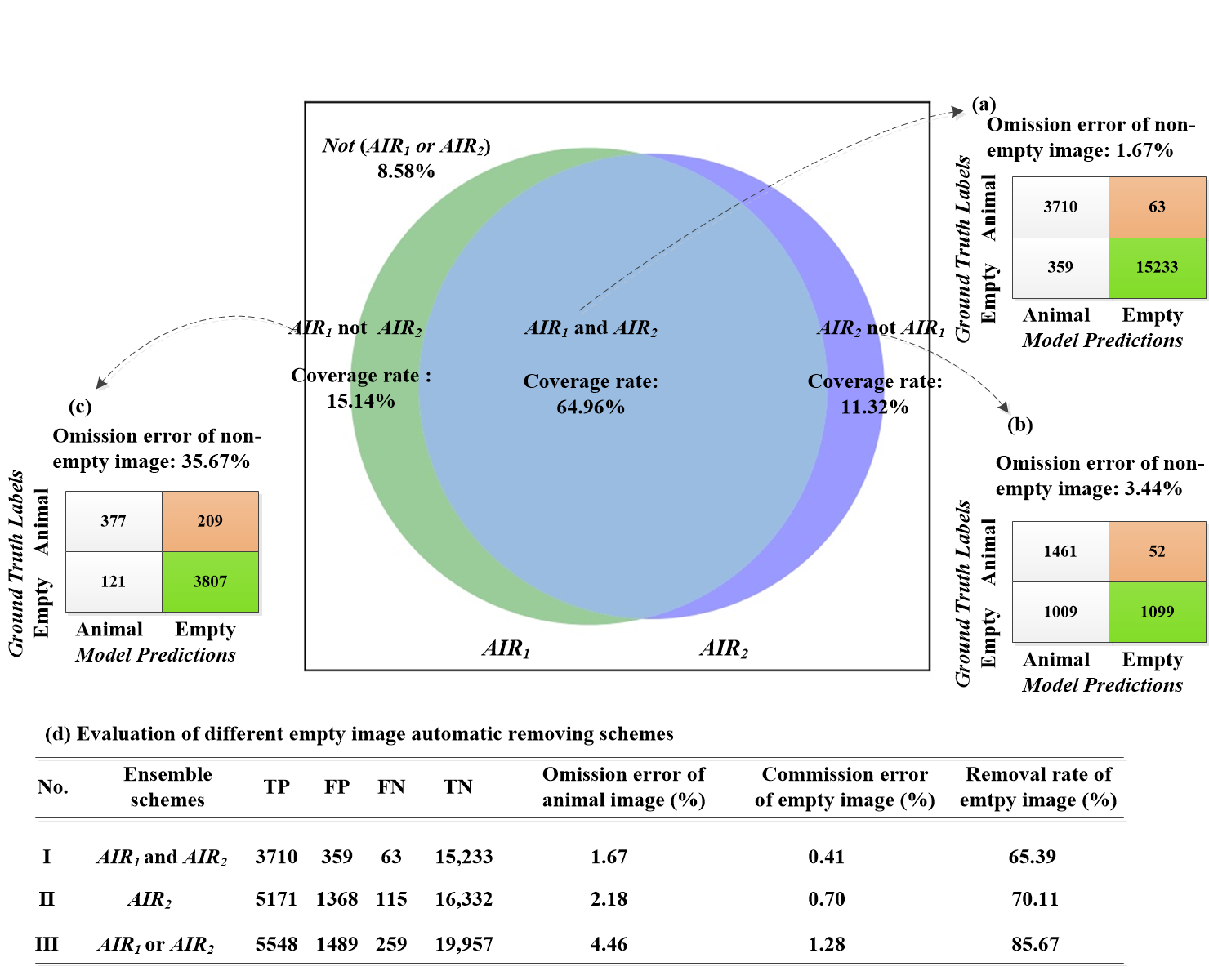


**Fig.S.10** Image-level results without confidence threshold of DCNN model

3.4.2 Capture event-level results

Figure S.11 shows the capture event-level results of the ensemble model without the confidence threshold. The enhanced ensemble model *AIR* (i.e., *AIR_1_* *and AIR_2_*) that did not set the confidence threshold of DCNN models can automatically identify and filter 61.30% of capture events. The omission error of non-empty event, commission error of empty event, and removal rate of empty event were 1.05%, 0.41%, and 56.95%, respectively. The ensemble model mistakenly removed 18 non-empty capture events. The complementary ensemble model *AIR* (i.e., *AIR_1_* or *AIR_2_*) that did not set the confidence threshold of DCNN models can automatically identify and filter 89.28% of capture events. The omission error of non-empty event, commission error of empty event, and removal rate of empty event were 3.44%, 1.21%, and 56.95%, respectively. The ensemble model mistakenly removed 78 non-empty capture events.


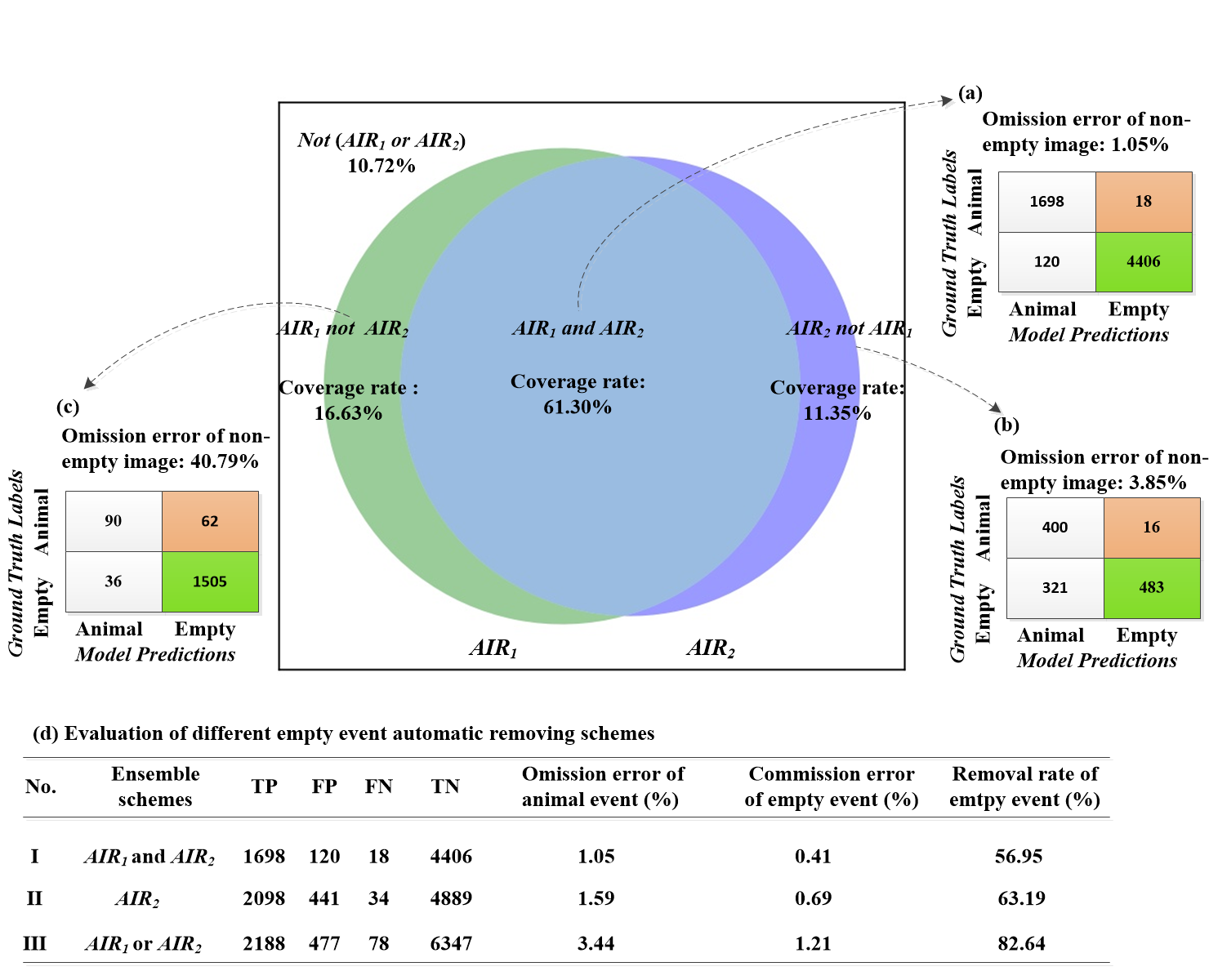


**Fig.S.11** Event-level results without confidence threshold of DCNN model

**3.5 Examples of missing images**

- Figure S.12 shows the images missed by the enhance ensemble model with a 95% confidence threshold.
- Figure S.13 shows the rare species images missed by the complementary ensemble model without the confidence threshold.


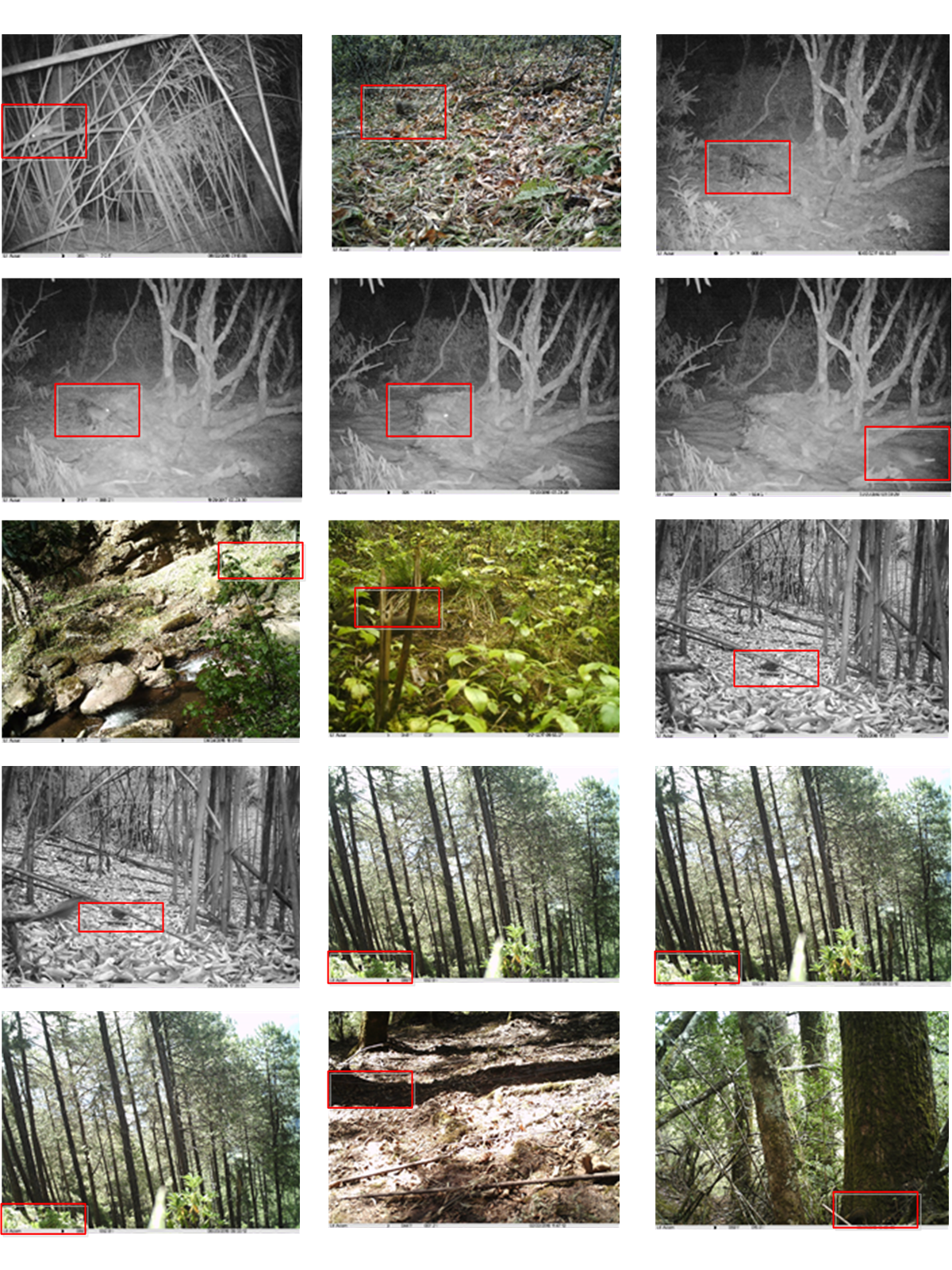


**Fig S.12** Image examples that were mistakenly removed by the enhance ensemble model (*AIR_1_* and *AIR_2_*) (with 95% confidence threshold)


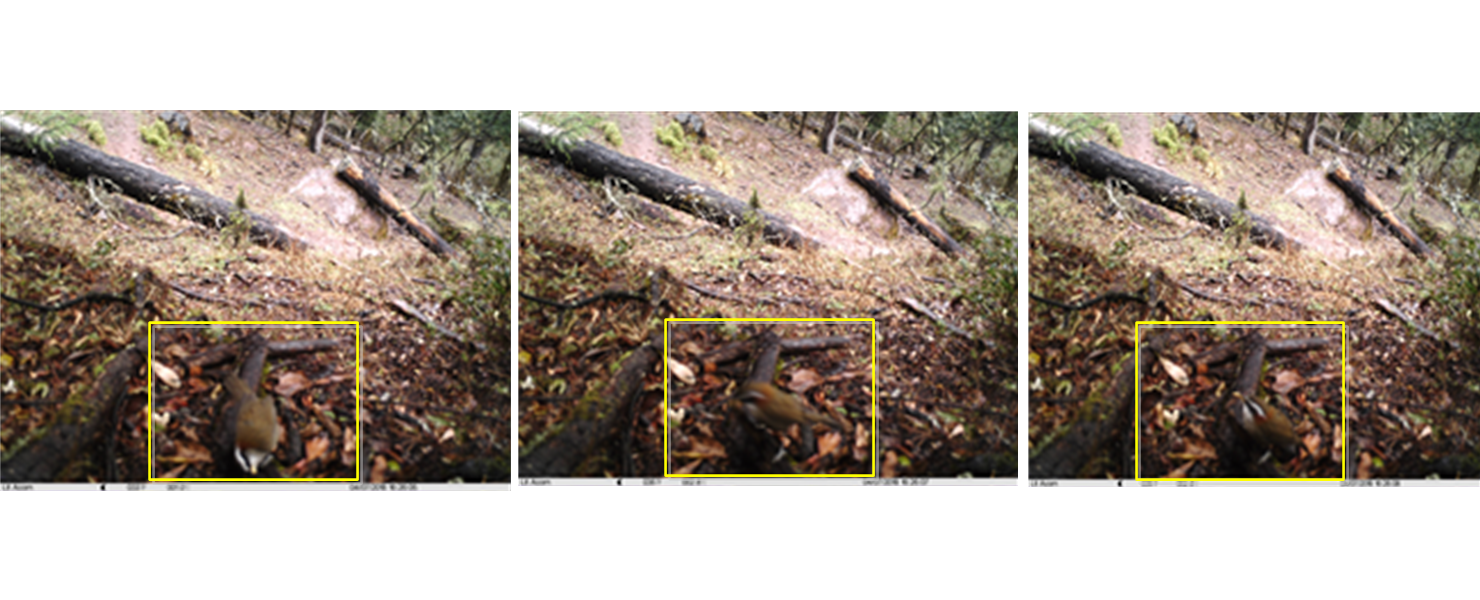


**Fig.S.13** Image examples of rare species missed by complementary ensemble method (*AIR_1_* or *AIR_2_*) without confidence threshold

**3.6 Confusion matrixes**

**Figure S.14** shows the confusion matrix of *Not (AIR_1_ or AIR_2_)*.

**Figure S.15** shows the confusion matrix of scheme *Ⅳ*.


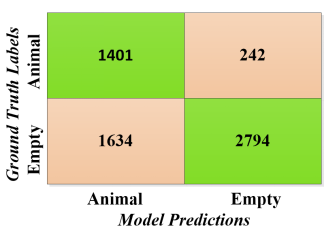


**Fig. S.14** Confusion matrix of *Not (AIR_1_ or AIR_2_)*.


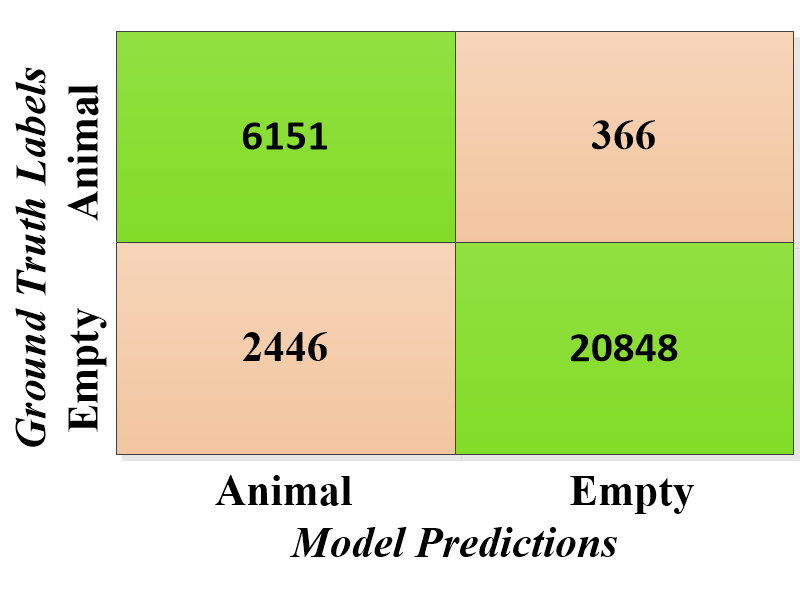


**Fig. S.15** Confusion matrix of the scheme *Ⅳ*

**4 Testing on the SS dataset**

**4.1 SS_S1_135 dataset**

The Snapshot Serengeti (SS) data set is the world’s largest camera-trap image dataset published to date, with 225 camera traps running continuously in Serengeti National Park, Tanzania, since 2011. The SS dataset contains camera trap images of multiple seasons. We selected the first 135 cameras (B04-P08) in the dataset of the first season (S1) and recorded them as the SS_S1_135. We extracted the first 332,192 images from the SS_S1_135 data set to construct the training sets and test set (Table S.9). We randomly split capture event and put all the images of the entire capture event into the training set or test set.

**TABLE S.7** The training sets and the test set of SS_S1_135

| Datasets | *Number of total images* | *Number of empty image* | *Number of non-empty images* | *Empty image percentage (%)* |
| --- | --- | --- | --- | --- |
| *SS_Train_1_* | 300,000 | 247,118 | 52,812 | 82.37 |
| *SS_Train_2_* | 105,624 | 52,812 | 52,812 | 50.00 |
| *SS_Test* | 32,192 | 26,621 | 5,570 | 82.69 |

**4.2 Experimental results on the SS_S1_135 dataset**

**4.2 The performance of the DCNN models under different confidence thresholds.**

4.2.1 Model confusion matrix of models with a 95% confidence threshold.


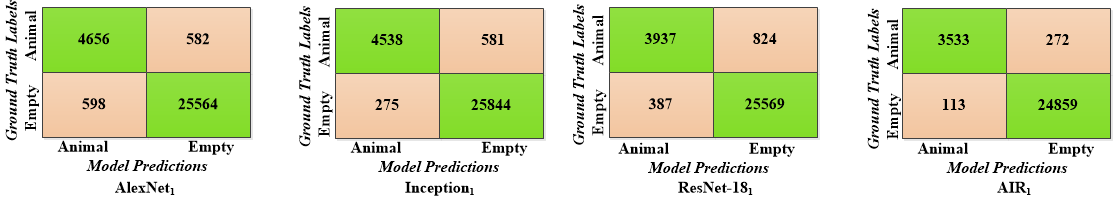


**Fig S.16** The confusion matrix of models on the unbalanced training set *SS_Train_1_* (with a 95% confidence threshold).


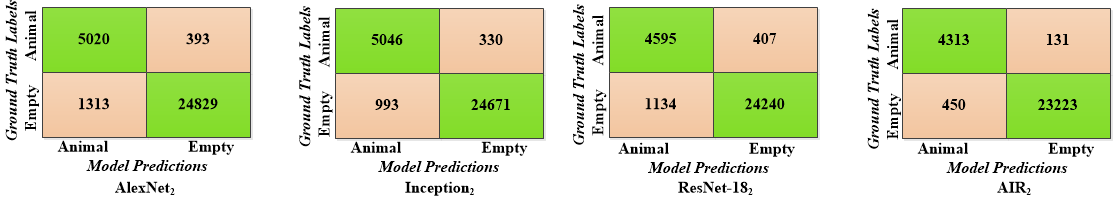


**Fig S.17** The confusion matrix of models on the unbalanced training set *SS_Train_2_* (with a 95% confidence threshold).

4.2.2 Model performance without confidence threshold (i.e., confidence=50%).

- **Table S.8** shows the performance of the individual DCNN models and the DCNN ensemble model *AIR_1_* (without the confidence threshold) using the imbalanced training set *Train_1_*. **Figure S.18** shows the corresponding confusion matrixes of models.
- **Table S.9** shows the performance of the individual DCNN models and the DCNN ensemble model *AIR_2_* (without the confidence threshold) using the imbalanced training set *Train_2_*. **Figure S.19** shows the corresponding confusion matrixes of models.

**TABLE S.8** The image-level results of model on the unbalanced training set *SS_Train_1_* (without the confidence threshold) ^*^

|  | ***E_ov_ (*%*)*** | ***E_oa_ (*%*)*** | ***E_ca_ (*%*)*** | ***E_ce_ (*%*)*** | ***R_re_ (*%*)*** |
| --- | --- | --- | --- | --- | --- |
| *AlexNet1* | 4.74 | 13.18 | 14.07 | 2.76 | 97.03 |
| *Inception_1_* | 3.84 | 14.09 | 8.61 | 2.91 | 98.31 |
| *ResNet_1_* | 5.72 | 21.51 | 12.80 | 4.41 | 97.59 |
| *Ensemble model (AIR_1_)* | 1.89 | 8.55 | 4.70 | 1.44 | 95.60 |

^*^ *E_ov_ was the overall error. E_oa_ was the omission error of animal images. E_ca_ was commission error of animal images image. E_ce_ was the commission error of empty images. R_re_ was the removal rate of empty images.*


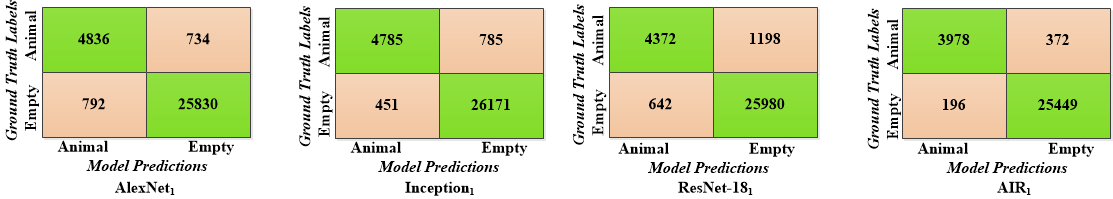


**Fig S.18** The confusion matrix of models on the unbalanced training set *SS_Train_1_* (without the confidence threshold).

**TABLE S.9** The image-level results of model on the unbalanced training set *SS_Train_2_* (without the confidence threshold) ^*^

|  | ***E_ov_ (*%*)*** | ***E_oa_ (*%*)*** | ***E_ca_ (*%*)*** | ***E_ce_ (*%*)*** | ***R_re_ (*%*)*** |
| --- | --- | --- | --- | --- | --- |
| *AlexNet1* | *6.19* | *8.26* | *23.07* | *1.80* | *94.25* |
| *Inception_1_* | *5.58* | *7.61* | *21.06* | *1.65* | *94.85* |
| *ResNet_1_* | *7.13* | *11.22* | *25.26* | *2.44* | *93.73* |
| *Ensemble model (AIR_1_)* | *2.97* | *4.20* | *12.84* | *0.83* | *90.62* |

^*^ *E_ov_ was the overall error. E_oa_ was the omission error of animal images. E_ca_ was commission error of animal images. E_ce_ was the commission error of empty images. R_re_ was the removal rate of empty images.*


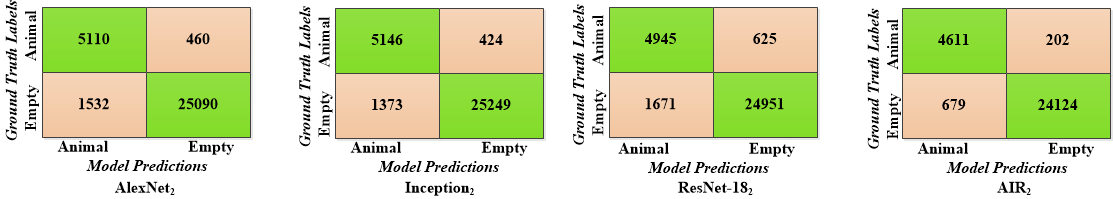


**Fig S.19** The confusion matrix of models on the unbalanced training set *SS_Train_2_* (without the confidence threshold).

4.2.3 Venn diagrams of ensemble model on SS_S1_135 dataset

- **Figure S.20** shows the image-level results of the ensemble model with a 95% the confidence threshold.
- **Figure S.21** shows the image-level results of the ensemble model without the confidence threshold.


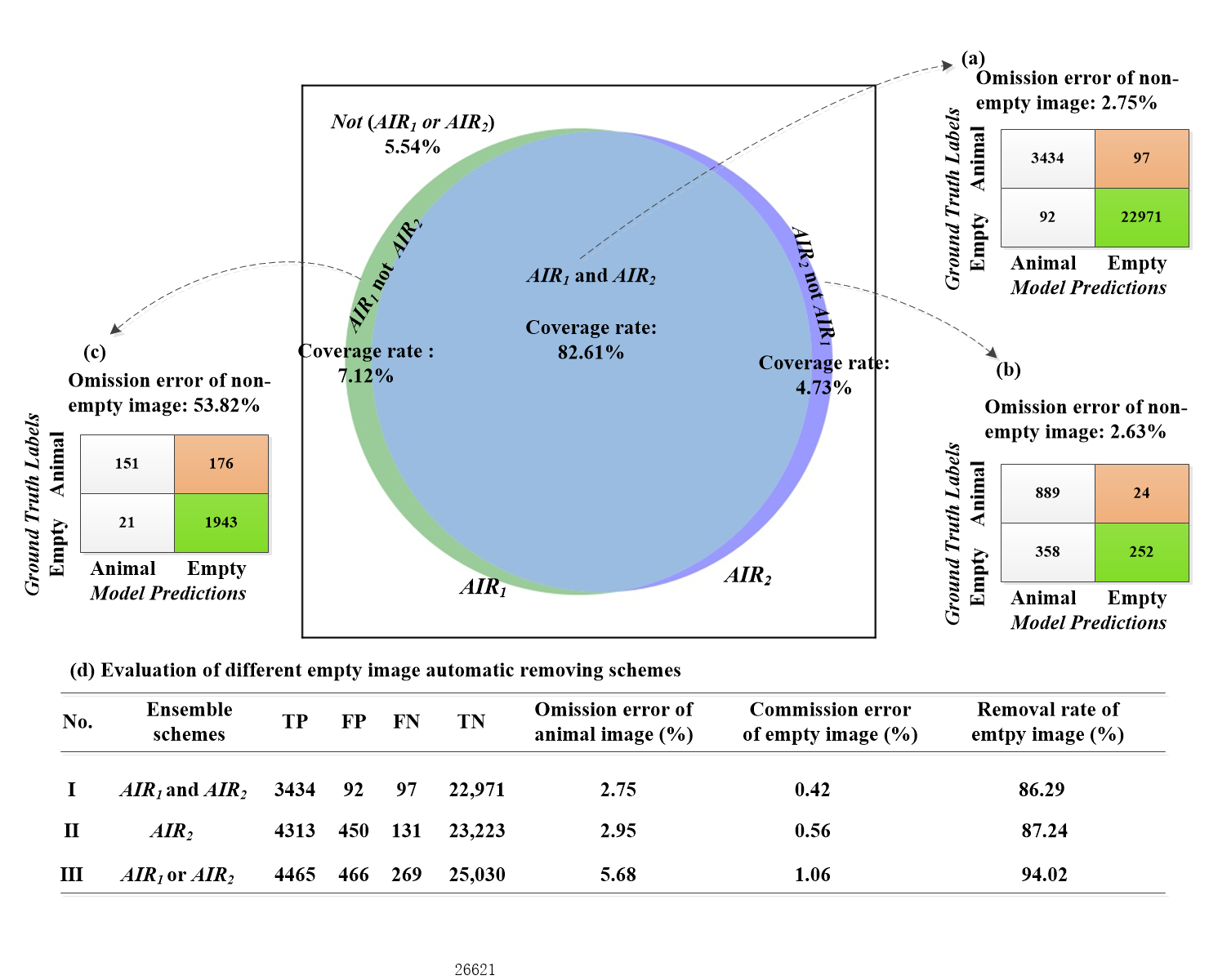


**Fig.S.20** Image-level experimental results on SS_S1_135 (with 95% confidence of DCNN models)


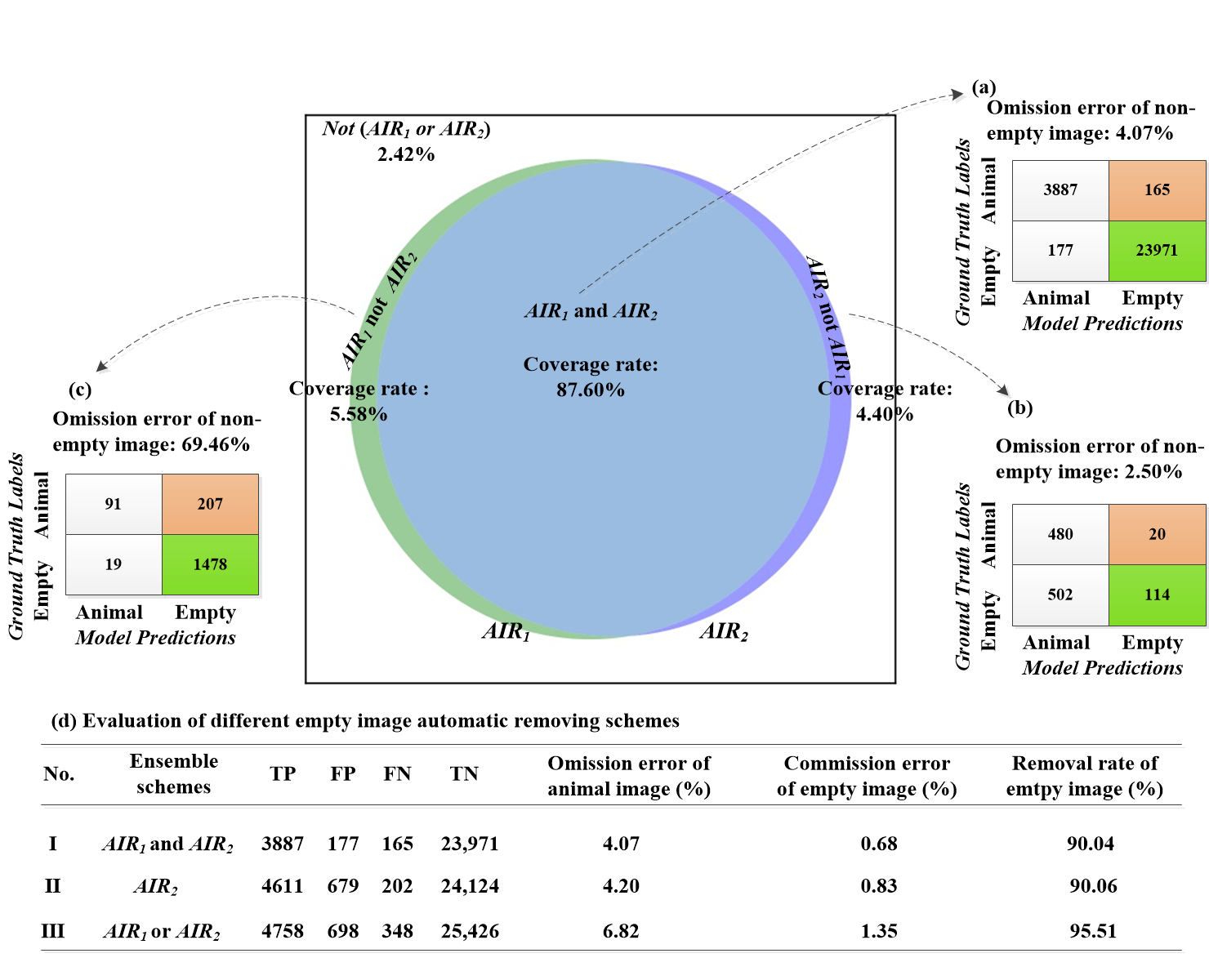


**Fig.S.21** Image-level results on SS_S1_135 (without confidence threshold of DCNN model)
